# Elucidating the Mechanism of Self-Healing in Hydrogel-Lead Halide Perovskite Composites for Use in Photovoltaic Devices

**DOI:** 10.1021/acsami.3c03359

**Published:** 2023-05-30

**Authors:** Dawei Zhao, Tom A. Flavell, Fahad Aljuaid, Stephen Edmondson, Ben F. Spencer, Alex S. Walton, Andrew G. Thomas, Wendy R. Flavell

**Affiliations:** † Photon Science Institute, University of Manchester, Oxford Road, Manchester M13 9PL, U.K.; ‡ Department of Materials, University of Manchester, Oxford Road, Manchester M13 9PL, U.K.; § Department of Physics and Astronomy, University of Manchester, Oxford Road, Manchester M13 9PL, U.K.; ∥ Henry Royce Institute, University of Manchester, Oxford Road, Manchester M13 9PL, U.K.; ⊥ Department of Chemistry, University of Manchester, Oxford Road, Manchester M13 9PL, U.K.

**Keywords:** perovskites, methylammonium lead iodide perovskites, polymer additives, near-ambient pressure X-ray photoelectron spectroscopy, hard X-ray photoelectron spectroscopy

## Abstract

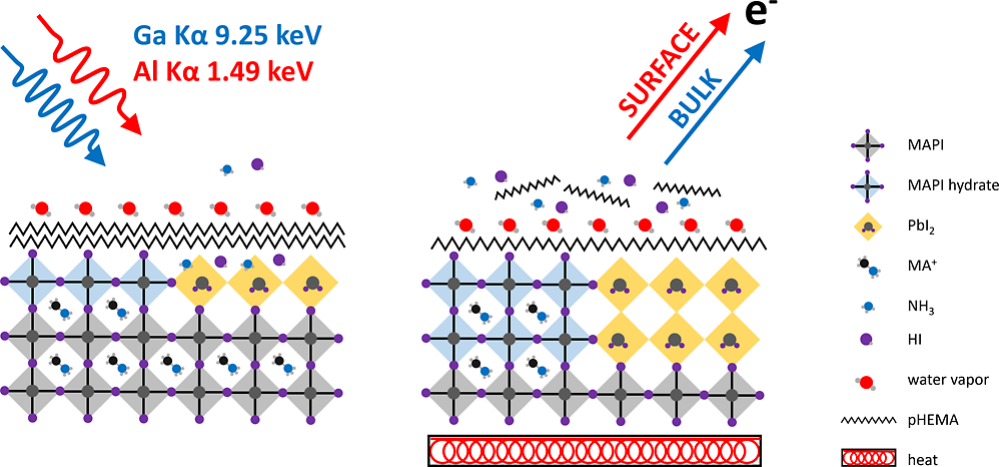

Since the emergence of organometal halide perovskite (OMP) solar cells, there has been growing interest in the benefits of incorporating polymer additives into the perovskite precursor, in terms of both photovoltaic device performance and perovskite stability. In addition, there is interest in the self-healing properties of polymer-incorporated OMPs, but the mechanisms behind these enhanced characteristics are still not fully understood. Here, we study the role of poly(2-hydroxyethyl methacrylate) (pHEMA) in improving the stability of methylammonium lead iodide (MAPI, CH_3_NH_3_PbI_3_) and determine a mechanism for the self-healing of the perovskite–polymer composite following exposure to atmospheres of differing relative humidity, using photoelectron spectroscopy. Varying concentrations of pHEMA (0–10 wt %) are incorporated into a PbI_2_ precursor solution during the conventional two-step fabrication method for producing MAPI. It is shown that the introduction of pHEMA results in high-quality MAPI films with increased grain size and reduced PbI_2_ concentration compared with pure MAPI films. Devices based on pHEMA-MAPI composites exhibit an improved photoelectric conversion efficiency of 17.8%, compared with 16.5% for a pure MAPI device. pHEMA-incorporated devices are found to retain 95.4% of the best efficiency after ageing for 1500 h in 35% RH, compared with 68.5% achieved from the pure MAPI device. The thermal and moisture tolerance of the resulting films is investigated using X-ray diffraction, in situ X-ray photoelectron spectroscopy (XPS), and hard XPS (HAXPES). It is found that exposing the pHEMA films to cycles of 70 and 20% relative humidity leads to a reversible degradation, via a self-healing process. Angle-resolved HAXPES depth-profiling using a non-destructive Ga Kα source shows that pHEMA is predominantly present at the surface with an effective thickness of ca. 3 nm. It is shown using XPS that this effective thickness reduces with increasing temperature. It is found that N is trapped in this surface layer of pHEMA, suggesting that N-containing moieties, produced during reaction with water at high humidity, are trapped in the pHEMA film and can be reincorporated into the perovskite when the humidity is reduced. XPS results also show that the inclusion of pHEMA enhances the thermal stability of MAPI under both UHV and 9 mbar water vapor pressure.

## 1 Introduction

Organometal halide perovskites (OHPs) are considered one of the most promising materials for use in next-generation solar cells. OHPs possess unique properties that make them extremely desirable as both absorbers and charge transporters in thin-film devices, including tunable band gaps, strong light-absorption coefficients, high defect tolerance, and low non-radiative recombination rates.^1−5^ Owing to these properties, 13 years on from the first use of OHP as a PV material, perovskite solar cells (PSCs) already boast photoelectric conversion efficiencies (PCE) as high as 25.7%,^6^ rivalling that of silicon-based devices, as well as considerably cheaper fabrication costs due to solution processability.^1,4,7,8^

However, the poor long-term stability of OHP materials, especially under ambient conditions, presents a major challenge in the drive for commercialization, as a device is required to maintain its efficiency for at least 25 years to be considered a viable power source for commercial and industrial applications.^2^ Intrinsic factors such as structural stability and extrinsic factors including moisture-, heat-, and light-induced degradation can negatively affect the optoelectronic properties of OHPs.^9−22^

Methylammonium lead iodide (MAPI, CH_3_NH_3_PbI_3_) is a commonly studied OHP due to ease of fabrication and favorable optoelectronic properties; however, irreversible compositional changes occur following its exposure to ambient air. The emission of ammonia (NH_3_) and hydrogen iodide (HI) gases leaves lead iodide (PbI_2_) and hydrocarbon chains (both non-photoactive) as the remaining decomposition products, as shown in eq 1(16)
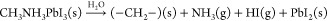
1

Thermal stress is known to instigate a similar degradation of MAPI to PbI_2_, with the complete loss of organic components from the surface, as shown in eq 2(23)
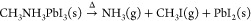
2

Control of the composition and morphology of perovskite thin films is key to enhancing both the stability and performance of devices. Defects are known to accelerate the decomposition of perovskites on exposure to atmospheric conditions, as well as through ion migration during device operation.^24−26^ Grain size has also been shown to influence perovskite degradation, with larger grains proving to be more robust when exposed to moisture.^27^

Various strategies to influence the morphology and improve the stability of perovskite films have been investigated, including compositional engineering such as cation mixing and surface passivation using coordination compounds.^2,28,29^ The introduction of additives to the perovskite precursor solution to influence crystal growth and passivate defect sites is seen as a promising and simple method of producing high-quality, resilient perovskite films.^30^

Several studies have explored the effects of incorporating polymers into perovskite thin films, both as an interfacial layer and as an additive. Hydrophobic polymer overlayers have been shown to improve the stability of perovskites under ambient conditions by preventing the intrusion of moisture; however, due to the hydrophilic nature of perovskites, it is difficult to form a uniform overlayer using hydrophobic polymers, which in turn can interfere with the contact between the perovskite absorber and the charge transport layer, negatively impacting charge transfer in devices.^31−33^ The inclusion of various polymers as additives in perovskite films has been shown to increase crystal grain size, passivate defect sites and improve film quality, resulting in enhanced device performance and stability.^30,34−36^

The polymer poly(2-hydroxyethyl methacrylate) (pHEMA) is unstudied as an additive for perovskite thin films and has several properties that make it an attractive candidate. pHEMA is a hydrophilic polymer and forms a cross-linked polymer network in water called a hydrogel. Other water-soluble polymer additives have been shown to act as templates to control perovskite nucleation and crystal growth, as well as passivate defects and inhibit ion migration.^35,37−42^ Hydrophilic additives are also beneficial for device performance, as contact is increased between the hydrophilic perovskite and charge transport layer, reducing energy loss at the interface.^30^ The hydrophilic polymer polyethylene glycol (PEG) has previously been shown to enhance the moisture tolerance of MAPbCl_3_ when added to the perovskite precursor during a one-step fabrication process.^35^ Devices based on this material were shown to recover rapidly following the removal of water vapor; however, no atomic scale investigation of the interfaces between components has been made elucidate this “self-healing” mechanism.^35,43,44^ The strong ability to absorb and retain water exhibited by pHEMA, central to its use as a contact lens material, may prevent moisture from interacting with the perovskite, thus enhancing the stability of the material.^45,46^ Additionally, pHEMA releases degradation products as it thermally decomposes, which are expected to escape during the annealing stages of MAPI fabrication.^47^ This is conducive to the formation of porous PbI_2_ during the conventional two-step perovskite fabrication approach, which would encourage the release of solvents and the incorporation of methylammonium iodide (MAI, CH_3_NH_3_I), resulting in improved film quality and stability.^48,49^

In this work, we investigate the effects of pHEMA additive on the stability of MAPI films and on the PCE of the corresponding devices. We illustrate that the incorporation of pHEMA produces high-quality MAPI thin films with large grain size and reduced uncovered PbI_2_ concentration, leading to devices with higher PCEs than those based on pure MAPI. However, the focus of this study is to better understand the mechanisms behind the enhanced stability of hydrogel-lead halide perovskite composites. Using a combination of in situ X-ray photoelectron spectroscopy (XPS) and non-destructive depth-profiling hard XPS (HAXPES, Ga Kα), supplemented by inelastic background analysis, we provide the most comprehensive surface stability study of hydrophilic polymer-MAPI composites to date. These data, in combination with X-ray diffraction (XRD) results, demonstrate the “self-healing” of pHEMA-MAPI composite films following exposure to water vapor. For the first time, we demonstrate the trapping of the decomposition residues of MA^+^ at the surfaces of the perovskite grains by the polymer, allowing facile self-healing. These findings support the mechanism proposed by Zhao et al., and provide new insights into the role of temperature during the self-healing process.^35^

## 2 Materials and Methods

### 2.1 Materials

Indium tin oxide substrates (ITO), methylammonium iodide (MAI, 98%), and 2,2′,7,7′-tetrakis(*N*,*N*-di-*p*-methoxyphenylamine)-9,9′-spirobifluorene (Spiro-MeOTAD, 99%) were purchased from Ossila. Isopropyl alcohol (IPA, 99.7%), anhydrous dimethylformamide (DMF, 99.8%), dimethyl sulfoxide (DMSO, 99.8%), acetonitrile (99.8%), chlorobenzene (99.9%), 4-*tert*-butylpyridine (4-*t*BP, 96%), lithium bis(trifluoromethanesulfonyl)imide (LiTFSI, 99.95%), tris(2-(1*H*-pyrazol-1-yl)-4-*tert*-butylpyridine)-tris(bis(trifluoromethylsulfonyl)imide) (FK209, 98%), and pHEMA were purchased from Sigma-Aldrich. Tin (IV) oxide (SnO_2_), Hellmanex III detergent, and lead iodide (PbI_2_) were purchased from Alfa Aesar.

### 2.2 Sample Fabrication

ITO glass substrates were cleaned sequentially with a Hellmanex III detergent, deionized (DI) water, acetone, and 2-propanol under ultrasonication for 20 min and then treated in a UV–ozone cleaner for 20 min. 1 mL of SnO_2_ colloid dispersion (15% in water) was diluted with 3 mL of DI water to obtain a clear solution, which was then coated on the ITO substrate by spinning at 3000 rpm for 30 s and annealing at 150 °C for 1 h. The perovskite layer was fabricated by a two-step spin-coating method under ambient conditions. The PbI_2_ precursor solution was prepared by dissolving 1.0 M PbI_2_ in 1 mL of DMF/DMSO (9:1). A dense PbI_2_ film was deposited by spin-coating PbI_2_ precursor solution at 2500 rpm for 30 s, followed by annealing at 80 °C for 30 min. Mixed PbI_2_/pHEMA films were prepared by adding pHEMA to the PbI_2_ precursor solution. Blended solutions of pHEMA and PbI_2_ at concentrations of 0, 2.5, 5, and 10 wt % were then spin spin-coated at 2500 rpm for 30 s. Subsequently, an MAI solution (50 mg mL^–1^ in IPA) was spin-coated at 1500 rpm for 30 s onto the prepared PbI_2_ or PbI_2_/pHEMA films. The films were then annealed at 100 °C for 20 min on a hotplate. For device preparation, a solution of spiro-MeOTAD in chlorobenzene (72.3 mg mL^–1^) with 28.8 μL of 4-*t*BP, 17.5 μL of LiTFSI in acetonitrile (520 mg mL^–1^) and 9 μL of FK209 in acetonitrile (300 mg mL^–1^) was cooled and spin-coated at 2000 rpm for 30 s to obtain the hole transport layer. Finally, 100 nm of Ag, chosen due to its lower cost compared with Au, was thermally evaporated under vacuum as the cathode. The active area of the device was determined by a mask of 0.024 cm^2^.

### 2.3 Characterization

The current–voltage (*J*–*V*) characteristic curves were measured using a Keithley 2420 source meter under AM 1.5G illumination (100 mW cm^–2^). The photovoltaic parameters were all obtained by reverse scanning direction from 1.2 to −0.1 V, unless otherwise stated. Both systems were calibrated against a certified reference solar cell. Measurements of the solar cells were performed under an ambient atmosphere at room temperature (RT) without encapsulation. Absorption spectra were recorded with an ultraviolet–visible–near-infrared (UV–vis–NIR) spectrometer (Lambda 1050, PerkinElmer). Photoluminescence spectroscopy was carried out using a FLS980 spectrometer (Edinburgh Instruments), in which the excitation wavelength was 450 nm. The morphology was measured using a scanning electron microscope (Quanta 250). The crystal structure and phase of the perovskite were characterized using an X-ray diffractometer [Cu Kα radiation (D8 ADVANCE, Bruker)].

### 2.4 Storage and Ex Situ Humidity Control

To control and maintain the humidity, silica-gel desiccants and DI water were used in lidded containers for device storage. A hygrometer was enclosed in each container, and the amount of silica-gel desiccant or DI-water was controlled to reach and maintain the target relative humidity (RH). The humidity in the containers was closely monitored.

### 2.5 X-ray Photoelectron Spectroscopy

Thermal degradation under ultra-high vacuum (UHV, <10^–8^ mbar) conditions was measured by XPS using an ESCA 2SR high-throughput X-ray photoelectron spectrometer (Scienta Omicron GmbH). The instrument is equipped with a monochromated Al Kα (1486.6 eV, 20 mA emission at 300 W) source, an Argus CU multi-purpose hemispherical analyzer, and an electron flood gun for less conductive samples. Samples were heated using a resistive heating filament attached to the sample base plate, and spectral acquisition commenced once a stable temperature was reached. This temperature was then maintained throughout the spectral acquisition. Survey scans and high-resolution core-level spectra were obtained at pass energies of 100 and 50 eV, respectively. The binding energy (BE) of the spectra was calibrated to the N 1s peak of MAPI (402.6 eV), and when not possible (due to the degradation of the perovskite), the spectra were calibrated to the Pb 4f peak of metallic Pb (Pb^0^, 137 eV).^16,50,51^ In cases where both chemical environments were present, calibration to N 1s was found to be consistent with a Pb^0^ BE of 137.0 eV. XPS data were analyzed using CasaXPS software, in which a Shirley background and pseudo-Voigt peaks were fitted to core-level spectra. Surface stoichiometric ratios were calculated from the intensities of the fitted peaks, using relative sensitivity factors (RSFs) from the Scofield library.

### 2.6 Near-Ambient Pressure XPS

Thermal degradation at 9 mbar water vapor pressure was measured using a laboratory-based near-ambient pressure XPS (NAP-XPS) system (SPECS). The instrument is equipped with a monochromated Al Kα (1486.6 eV) source and PHOIBOS 150 NAP hemispherical analyzer fitted with a three-stage, differentially pumped electrostatic lens. Water vapor was introduced to the samples in a NAP gas cell at a pressure of 9 mbar, corresponding to a RH of ∼30%. Spectra were acquired under UHV conditions at RT (before water exposure), following the introduction of 9 mbar water vapor pressure and then at 100 and 150 °C, with 9 mbar pressure maintained. After exposure, measurements were taken at RT under UHV conditions, after pumping out the water vapor. Spectra were calibrated and processed as outlined for our XPS measurements; however, RSFs for the analyzer are not known under NAP conditions. Thus, surface stoichiometric ratios were only calculated at UHV (before and after exposure).

### 2.7 HAXPES

Angle-resolved HAXPES measurements were performed with a high-throughput HAXPES laboratory-based system (Scienta Omicron GmbH) under UHV conditions. The instrument is equipped with a Ga Kα metal jet X-ray source (Excillum, 9.25 keV), bespoke monochromator, and EW-4000 electron energy analyzer, which can measure photoelectrons with kinetic energy (KE) of up to 12 keV. Depth profiling was achieved by tilting the samples with respect to the analyzer normal and measuring at various emission angles (4–70°), with respect to the surface normal, thus reducing the sampling depth. Sampling depth, *d*_s_, was estimated using
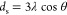
3where λ is the inelastic mean free path (IMFP) and θ is the electron emission angle relative to the surface normal.^52,53^ Stoichiometric ratios were calculated from the core-level peak intensities using RSFs from the Ga Kα library.^54^

### 2.8 Near-Edge X-ray Absorption Fine Structure and StoBe-deMon

Near-edge X-ray absorption fine structure (NEXAFS) measurements were performed at the flexible photoelectron spectroscopy (FlexPES) beamline, MAX IV Laboratory, Lund, Sweden. The beamline photon source is a linearly-polarizing undulator, which produces X-rays with an energy range of 40–1500 eV and a flux of 10^10^ photons s^–1^ at a photon energy of 1000 eV. The surface and material science branch permanent UHV end station consists of an analysis chamber equipped with a NEXAFS MCP detector at a base pressure of 3 × 10^–10^ mbar, two preparation chambers, and a fast entry chamber. For partial electron yield (PEY) measurements, a 260 V cutoff was applied.

Theoretical NEXAFS spectra were produced using density functional theory (DFT) calculations in the StoBe-deMon (ver. 3.3) software, for comparison with the experimental results.^55^ Avogadro (ver. 1.2.0) was used to optimize the structure of a HEMA molecule using molecular dynamics, the coordinates from which were input into the StoBe-deMon package. The carbon K-edge spectrum was constructed by summing the spectra simulated for each individual carbon atom. Each spectrum was broadened using Gaussian functions with linearly increasing full width half maxima (fwhm), from 0.7 at 290 eV to 12.0 at 310 eV, to account for the reduced lifetime of the σ* resonances.^56,57^

### 2.9 Inelastic Background Modeling

QUASES-Tougaard software was used to perform inelastic background modeling of XPS and HAXPES data, in which the built-in polymer inelastic cross-section model was employed.^58,59^ The IMFP at various energies was determined from the Tanuma–Powell–Penn formula (TPP-2M) to calculate the IMFP from a set of user-defined material parameters.^60,61^

## 3 Results

### 3.1 Fabrication of Dense PbI_2_, Porous PbI_2_, and MAPI Films

**Figure 1:**
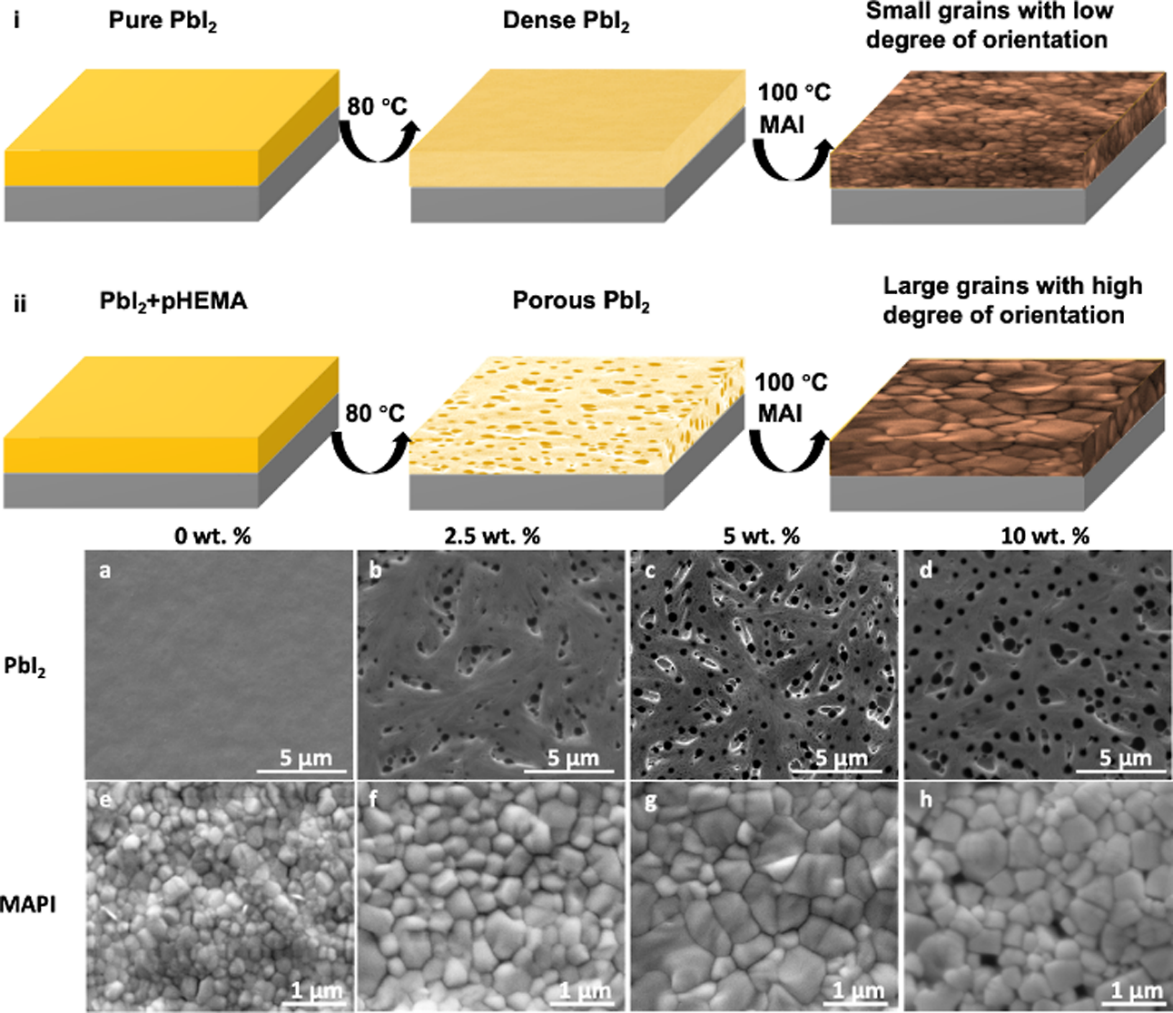
(i,ii) Schematic illustration of the fabrication procedure for pure PbI_2_ and pHEMA-incorporated PbI_2_ and the corresponding MAPI films. (a–d) SEM images of pure PbI_2_ (0 wt %) and pHEMA-incorporated PbI_2_ (2.5–10 wt %). (e–h) Corresponding MAPI films formed from the PbI_2_ layers.

Figure 1i,ii shows the schematic diagrams of the preparative route for dense PbI_2_ and pHEMA PbI_2_ and scanning electron microscopy images of the corresponding MAPI films. Typically, the PbI_2_ layer produced using the traditional two-step method is quite dense, making it difficult for MAI to fully penetrate, thereby affecting the MAPI conversion and reaction speed. As can be seen in Figure 1b–d, when pHEMA is added to the PbI_2_ and annealed on a hot plate, a porous PbI_2_ film is formed. The amount of pHEMA in PbI_2_ can be adjusted to control the pore formation of PbI_2_. This porous PbI_2_ can serve as a diffusion channel for MAI and appears to significantly improve the MAPI conversion and crystallization quality.

### 3.2 Near-Edge X-ray Absorption Fine Structure

NEXAFS measurements were performed to characterize the 0 wt % (pure MAPI) and 5 wt % pHEMA-MAPI films. C K-edge and N K-edge spectra from pristine samples are shown in Figure S1. Both spectra display characteristic peaks associated with MAPI bonding environments, and an additional peak associated with the C 1s to C=O π* transition within the pHEMA is present in the 5 wt % C K-edge, consistent with DFT simulations for HEMA.^55,62−64^ These results confirm that following the formation of the MAPI film, some of the pHEMA additive remains intact as part of a mixed system.

### 3.3 Morphology, Device Structure, and Performance

As mentioned above, the SEM images shown in Figure 1a–d indicate that the surface of the PbI_2_ prepared by the traditional method is very uniform with no visible pore holes, but addition of pHEMA leads to a porous PbI_2_ film. The pure MAPI films (shown in Figure 1e) have small grains and an uneven size, suggesting poor incorporation of MAI into the films. The 2.5 wt % pHEMA-PbI_2_ films on the other hand, lead to a MAPI film with a slightly enlarged grain size (Figure 1f) compared to the pure MAPI film. When the amount of pHEMA was increased to 5 wt %, more uniformly distributed pores are observed on the surface of the PbI_2_ (Figure 1c), and the grains of the corresponding MAPI films (Figure 1g) are further enlarged. When 10 wt % pHEMA was added to the PbI_2_, the pores in the PbI_2_ films (Figure 1d) were denser and larger as more pHEMA decomposed under heating. This appears to lead to the formation of pores in the MAPI films (Figure 1h), which affects the compactness of the MAPI films and is likely to affect charge transport in the film.^65^

**Figure 2:**
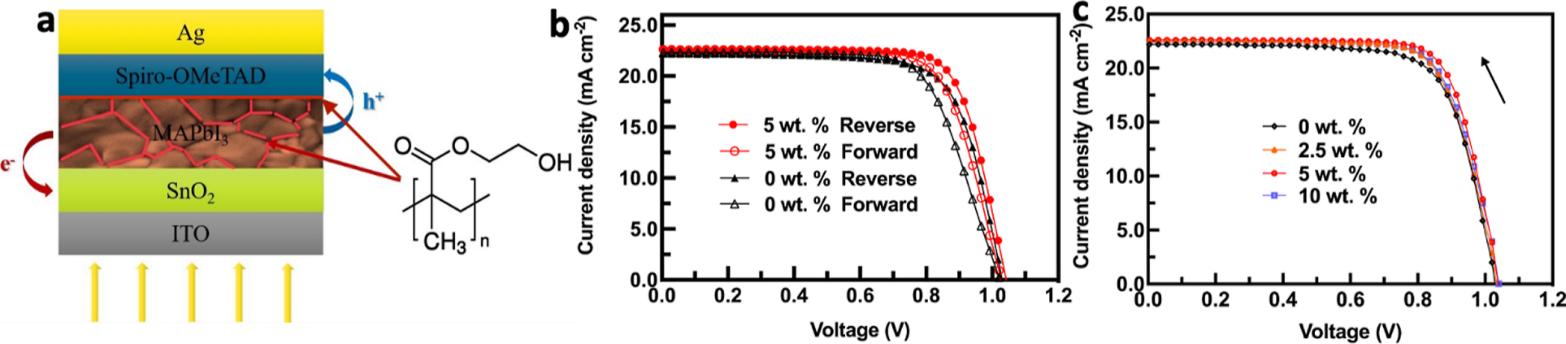
(a) Schematic diagram of the PSC with pHEMA and the chemical structure of pHEMA. (b) *J*–*V* curve of PSCs with 0 and 5 wt % pHEMA by reverse and forward scans. (c) Reverse scan *J*–*V* curves of PSCs with various concentrations of pHEMA (0–10 wt %).

Figure 2a shows the structure of a pHEMA-incorporated device. The introduction of pHEMA to the MAPI precursor may allow the polymer to penetrate the grain boundaries of the perovskite. This assumption will be discussed in more detail below. Figure S2a shows the UV–vis–NIR absorbance spectra of MAPI films prepared with various concentrations of pHEMA. Within the 500–750 nm range, the absorbance of pHEMA-incorporated MAPI films is higher than the 0 wt % sample, particularly at 5 and 10 wt % pHEMA concentration, which can be attributed to the increase in grain size demonstrated by SEM images in Figure 1e–h.^66^ Cross-sectional SEM images (Figure S3) demonstrate that film thickness remains constant within error for all pHEMA concentrations (500 ± 20 nm) and, therefore, has no influence on the absorbance of these samples. As shown in Figure S2b, the PL intensity of the samples modified with pHEMA is also enhanced, reaching a maximum at 5 wt %. The results are consistent with the UV–vis–NIR spectra and suggest that pHEMA helps to reduce the density of defect states in MAPI, thereby reducing the energy loss caused by non-radiative recombination.

Figure S2c shows the XRD patterns of MAPI films based on different concentrations of pHEMA. The PbI_2_ characteristic (001) reflection appears at 12.7°. The characteristic peaks of MAPI appear at 14.2, 28.5, and 31.9°, assigned to the (110), (220), and (310) reflections, respectively. The MAPI prepared without pHEMA has a strong reflection due to PbI_2_, indicating that there is significant residual PbI_2_ in the MAPI film. However, the PbI_2_ peak of the pHEMA-incorporated MAPI films is much weaker, suggesting that the inclusion of pHEMA results in a more complete reaction between the PbI_2_ and the MAI. This, along with the SEM data above, suggest that addition of pHEMA leads to an improved perovskite film quality compared to the pure PbI_2_. The reduction in (110) and increase in (310) MAPI reflections at 10 wt % indicates that at high loading, pHEMA changes the preferred orientation of the perovskite. This is reflected in the corresponding SEM image shown in Figure 1h, which reveals the emergence of pores and uneven grain sizes at 10 wt %.

Figure 2b,c demonstrates the positive effect of the porous PbI_2_ structure achieved through incorporation of pHEMA on the performance of PSC devices, with a champion PCE of 17.8% achieved with the 5 wt % device, compared with 16.5% achieved with the 0 wt % device, as shown in Table 1. A slight increase in short circuit current density (*J*_SC_) and open circuit voltage (*V*_OC_) was displayed by the 5 wt % device due to the higher light absorption and PL intensity of the pHEMA-incorporated film (see Figure S2), as well as an improvement in fill-factor (FF), implying an increase in the maximum power point. Charge carrier recombination rate and mobility have a strong influence on FF; thus, the inclusion of the insulating pHEMA additive could be expected to have a negative effect on FF and, therefore, on device performance.^67−70^ However, also key to reducing carrier recombination is the passivation of defects that act as electron or hole traps.^70−72^ As shown in Figures 1 and S2c, the inclusion of pHEMA results in MAPI films with larger grain size and reduced PbI_2_ concentration; therefore, we suggest that the negative impact of pHEMA on charge transport is offset by the passivation of defects, resulting in a slight enhancement of device performance. The PCE of devices based on different concentrations of pHEMA were also compared, and it was found that the efficiency of pHEMA-incorporated devices reaches a maximum at a concentration of 5 wt %. The photovoltaic parameters of these PSCs are recorded in Table S1.

**Table 1:** Photovoltaic Parameters of PSCs with 0 and 5 wt % pHEMA^a^

sample	scan direction	*J*_SC_ (mA cm^–2^)	*V*_OC_ (V)	FF (%)	PCE (%)
0 wt %	R (average)	22.3 ± 0.1	1.02 ± 0.02	72.5 ± 0.3	16.4 ± 0.2
	R (champion)	22.2	1.03	72.3	16.5
	F (champion)	22.3	1.02	69.1	15.7
5 wt %	R (average)	22.6 ± 0.2	1.03 ± 0.02	76.3 ± 0.8	17.8 ± 0.1
	R (champion)	22.6	1.04	75.7	17.8
	F (champion)	22.6	1.02	73.0	16.9

a Each average parameter comes from a set of 10 devices; R = reverse scan direction, F = forward scan direction.

### 3.4 Evolution of Photovoltaic Performance in Humid Environments

**Figure 3:**
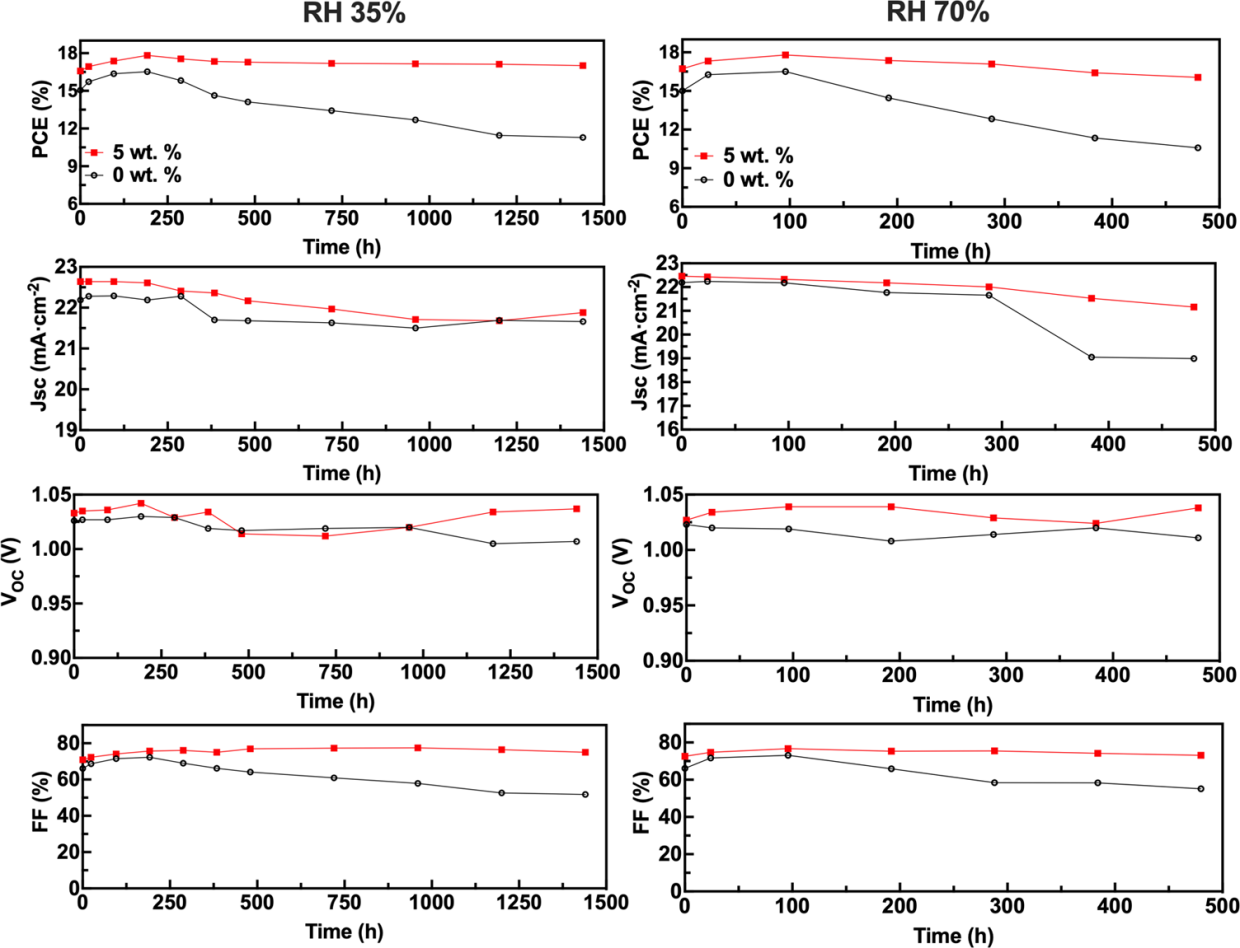
Time evolution of the photovoltaic performance of PSCs manufactured with 0 and 5 wt % pHEMA. The devices were held at 35% RH (left) and 70% RH (right).

In order to monitor the stability of the devices in different humidities, PSCs based on PbI_2_ synthesized with and without pHEMA were aged for nearly 1500 h in a 35% RH environment, and the device performance was continuously tested under these conditions. The variation in the photovoltaic parameters of the devices is shown in Figure 3 (left). The device manufactured with pHEMA still maintains 95.4% of the best efficiency after aging for 1500 h in 35% RH, showing excellent stability compared to the reference sample, which only maintained 68.5% of its maximum. When the humidity was further increased to 70% RH, the difference in the stability of PSCs manufactured via the two routes was more obvious [Figure 3 (right)]. After 500 h of aging in 70% RH, the device manufactured with pHEMA still retains 90.2% of its best efficiency, while the pure MAPI device only retained 64.2% of its maximum. The photovoltaic parameters of these devices are recorded in Tables S2 and S3. We suggest that the improved stability in the presence of moisture is in part due to the porous structure of PbI_2_ being conducive to the release of DMF and DMSO solvent, which is beneficial to the improvement of the humidity stability of the device.^49^ We also attribute the enhanced stability to the passivation of defects and added protection from water vapor provided by the pHEMA.^73^Figure 3 highlights that FF is the main source of PCE loss for the 0 wt % device, likely due to increased degradation resulting in a higher density of defects in the MAPI film.^70−72^ All of the above features appear to improve the long-term stability of the device under high humidity.

### 3.5 XRD Patterns and UV–vis–NIR Spectra during Degradation

To monitor the degradation of the MAPI films more accurately, XRD was used to characterize the changes of crystalline phase under different humidities [Figure S4 (left)]. At 35% RH, the characteristic (001) reflection of PbI_2_ (12.7°) in the pure MAPI film gradually increases with aging time, indicating that the degradation becomes gradually more serious, which is consistent with observations of the MAPI film appearance, shown in Figure S5. At 70% RH, the intensity of the (001) reflection of PbI_2_ from the pure MAPI sample was very significant by 7 days. However, when 2.5 and 5 wt % pHEMA was introduced, a significant PbI_2_ (001) reflection was only observed after 21 days, effectively delaying the decay of the MAPI film. It is worth noting that when 10 wt % pHEMA was introduced, there was no significant production of PbI_2_ throughout the observation period. The excellent water vapor stability of the pHEMA-incorporated MAPI is likely to be associated with the strongly hydrophilic nature of pHEMA, which is a hydrogel material. It can reversibly absorb water, which may reduce the likelihood of water vapor coming into contact with the perovskite.^74^ This hypothesis is explored further in Section 3.8. In addition, it is also possible that pHEMA filling at the grain boundaries of MAPI means that defects are passivated, while the porous structure of the PbI_2_ film used to synthesize the MAPI films promotes DMF solvent release.

It is clear from the XRD results that the effects of degradation are much more significant at 70% RH than at 35% RH. UV–vis–NIR spectra were used to further probe the effect of the PbI_2_ porous structure and pHEMA gel network on the light absorption of the MAPI films. It can be seen from Figure S4 (right) that at 35% RH, the light absorption of the MAPI films did not change significantly, which may be because the humidity was too low to cause serious degradation of the MAPI film. At 70% RH, the absorbance of the pure MAPI film dropped significantly by 7 days, and further dropped to about half of the fresh sample by 10 days, indicating its poor stability under high humidity. The pHEMA-incorporated MAPI films all showed a drop in absorbance at wavelengths below 700 nm, which is consistent with the observations of the XRD results and the film appearance, as shown in Figure S5. The protective effect of the hydrogel network on the MAPI film is discussed further in Section 3.9.

### 3.6 Thermal Decomposition under UHV Conditions

**Figure 4:**
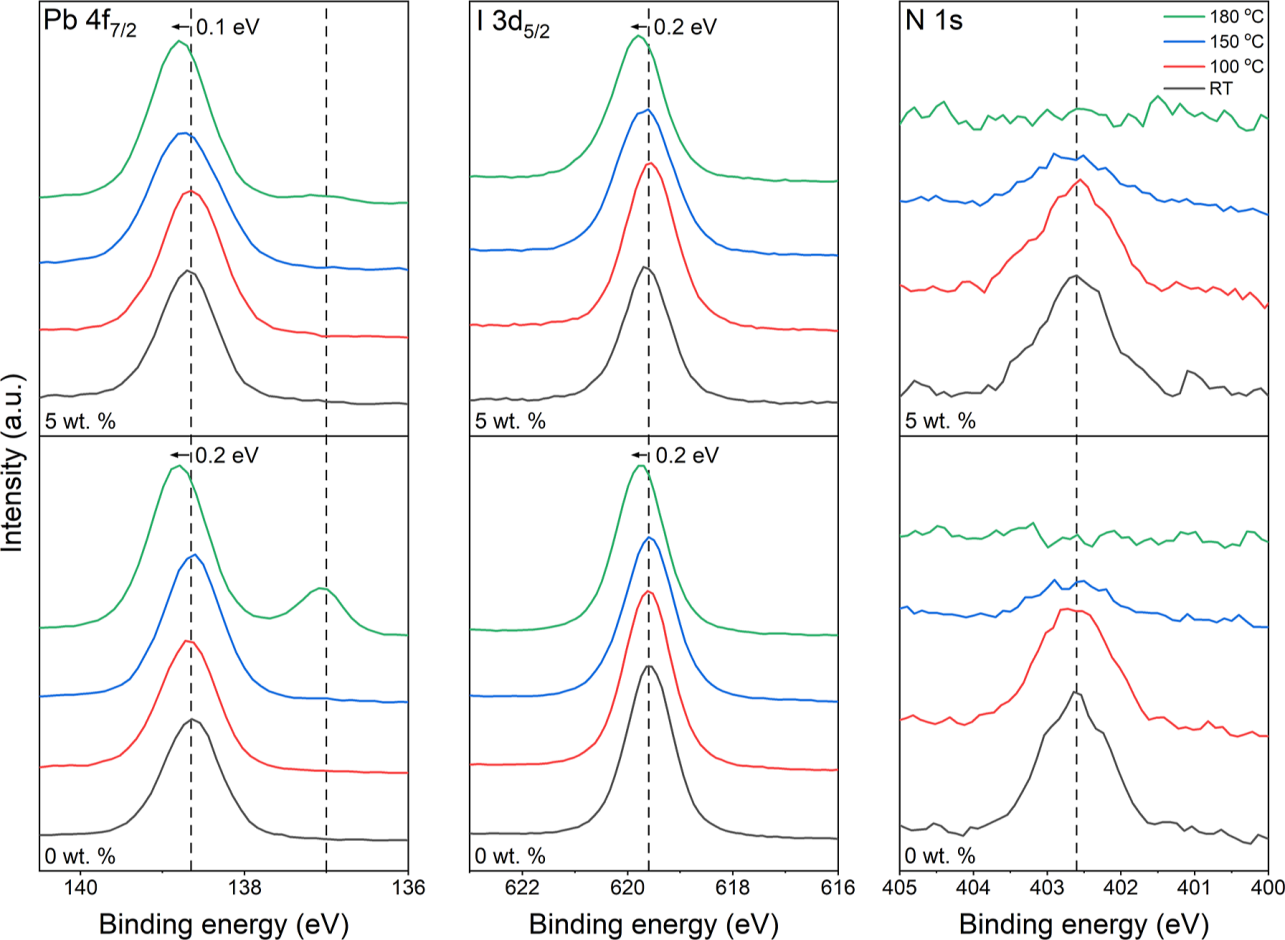
Pb 4f_7/2_, I 3d_5/2_, and N 1s (left to right) high-resolution core-level XPS spectra of the 0 wt % (bottom) and 5 wt % (top) films, measured at RT, 100, 150, and 180 °C. Initial peak positions are indicated by the dashed lines and the final shift is noted (as measured at 180 °C).

In order to probe the degradation process at the atomic scale and, in particular, to distinguish between the effects induced by moisture and those caused by heating, XPS was employed under controlled conditions. First, the thermal decomposition of a 0 wt % (pure MAPI) and 5 wt % pHEMA-MAPI film was monitored using XPS at four different temperatures (RT, 100, 150, and 180 °C) under UHV conditions. Pb 4f_7/2_, I 3d_5/2_, and N 1s high-resolution core-level XPS spectra are displayed in Figure 4.

At RT, the Pb 4f_7/2_ spectra consist of a single characteristic peak at 138.6 ± 0.1 and 138.7 ± 0.1 eV BE for the 0 and 5 wt % films, respectively. This is in excellent agreement with previous reports for the Pb^2+^ chemical state in MAPI.^16,50,51^ A BE shift is observed during the heating process, resulting in a final displacement of +0.2 and +0.1 eV at 180 °C of the 0 and 5 wt % films, respectively. This is consistent with the decomposition of the perovskite to PbI_2_.^16,51^ Also prominent in the 0 wt % Pb 4f_7/2_ spectrum at 180 °C is a peak at 137.0 ± 0.1 eV, attributed to Pb^0^ formation due to thermally induced PbI_2_ decomposition.^16,51,53^ A Pb^0^ peak at 137.0 ± 0.1 eV is also present in the 5 wt % Pb 4f_7/2_ spectrum at 180 °C; however, the intensity is significantly lower than that measured from the 0 wt % film.

I 3d_5/2_ spectra recorded at RT display a single characteristic peak at 619.6 ± 0.1 eV BE for both samples, in line with previous XPS studies of MAPI.^16,50,51^ A similar BE shift of +0.2 eV is observed for both samples following heating to 180 °C, consistent with the shifts observed in the Pb 4f_7/2_ spectra due to PbI_2_ formation as the MAPI degrades. PbI_2_ formation is understood to occur as the methylammonium cation (MA^+^) decomposes to ammonia gas, resulting in a loss of nitrogen from the surface.^9^ This process is highlighted in the N 1s spectra, which display a single peak at 402.6 ± 0.1 eV BE at RT for both samples, attributed to MA^+^, the intensities of which reduce to zero at 180 °C. This indicates the complete degradation the organic cation, with all nitrogen lost from the surface in the form of ammonia gas.^16^

**Figure 5:**
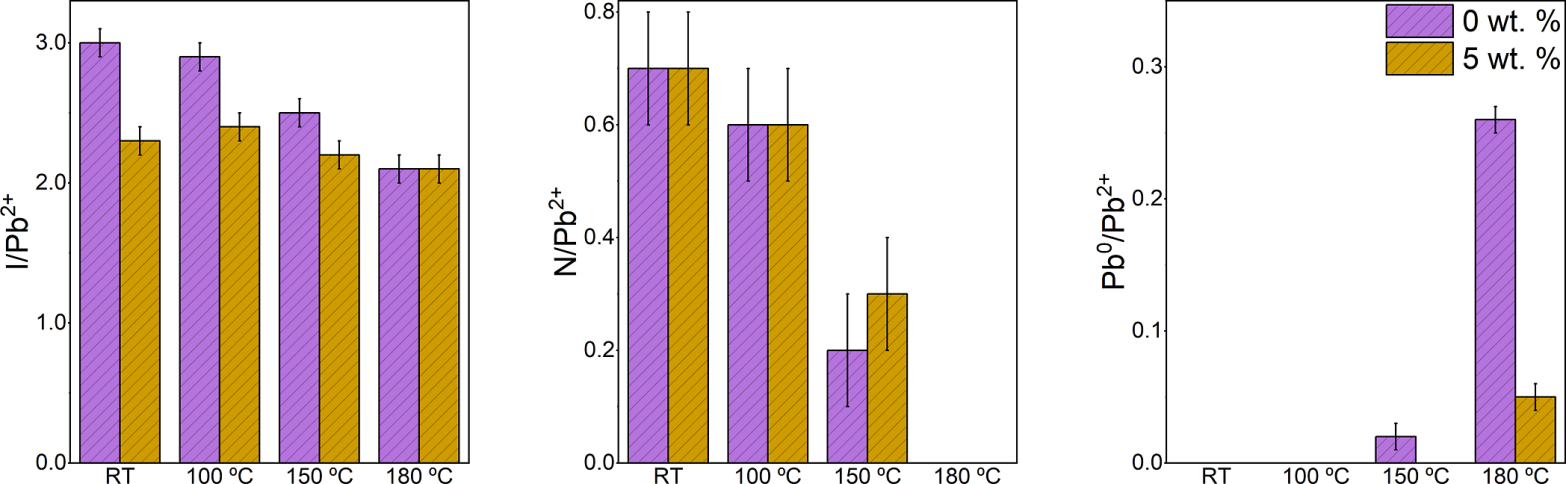
Surface stoichiometry of the 0 and 5 wt % films as a function of sample temperature, determined from fitted XPS peak intensities. I, N, and Pb^0^ concentrations with respect to Pb^2+^ (left to right) are displayed. The nominal stoichiometry of MAPI is 1:3:1 (Pb^2+^:I:N). Values are shown in Tables S4 and S5.

Elemental concentrations were determined from peaks fitted to the high-resolution core-level XPS spectra, the evolutions of which are displayed in Figure 5. A significant difference in the I/Pb^2+^ ratio at RT is noted between the two samples. The 0 wt % film displays a ratio of 3.0 ± 0.1, consistent with the nominal stoichiometry of pristine MAPI, whereas the 5 wt % film has a lower ratio of 2.3 ± 0.1. Throughout the heating regime, the 5 wt % I/Pb^2+^ ratio undergoes little change, resulting in a final ratio of 2.1 ± 0.1 at 180 °C. Conversely, the 0 wt % film shows a larger change in the I/Pb^2+^ ratio, which also falls to 2.1 ± 0.1 at 180 °C. A similar change of nitrogen concentration is observed in both samples, with the N/Pb^2+^ ratio reducing from 0.7 ± 0.1 at RT to zero at 180 °C. Significantly, Pb^0^ formation appears to be greatly reduced in the 5 wt % film, which exhibits a Pb^0^/Pb^2+^ ratio of only 0.05 ± 0.01 at 180 °C. In comparison, the 0 wt % film displays evidence of Pb^0^ formation at 150 °C and exhibits a ratio of 0.26 ± 0.01 at 180 °C. C 1s and O 1s high-resolution core-level spectra at RT are shown in Figure S6.

Atomic ratios of C/Pb^2+^ were calculated from the intensities of peaks fitted to the core levels displayed in Figure S6. In this case, the total intensity of the C 1s spectra was taken. For the 0 wt % film, this was found to be 4.9 ± 0.1, greater than the nominal value of 1 due to surface carbon contamination. The ratio for the 5 wt % film was found to be 33.5 ± 0.1, much larger than the nominal C/Pb^2+^ ratio for 5 wt % of pHEMA, calculated as 3.5. Assuming similar levels of adventitious carbon contamination for both samples, this value suggests that a much larger concentration of pHEMA is present at the surface of the sample than is incorporated into the bulk. Atomic ratios of O/Pb^2+^ were similarly calculated, taking the total intensity of the O 1s spectra. This ratio was found to be 3.7 ± 0.1 for the 0 wt % film, where the O is assumed to originate entirely from the ITO substrate and adsorbed oxygen-containing contaminants such as water and CO_2_. For the 5 wt % film, the O/Pb^2+^ ratio was found to be 41.6 ± 0.1, significantly larger than the nominal value of 2.2. Again, this points to a much larger concentration of pHEMA at the surface of the sample than in the bulk.

**Figure 6:**
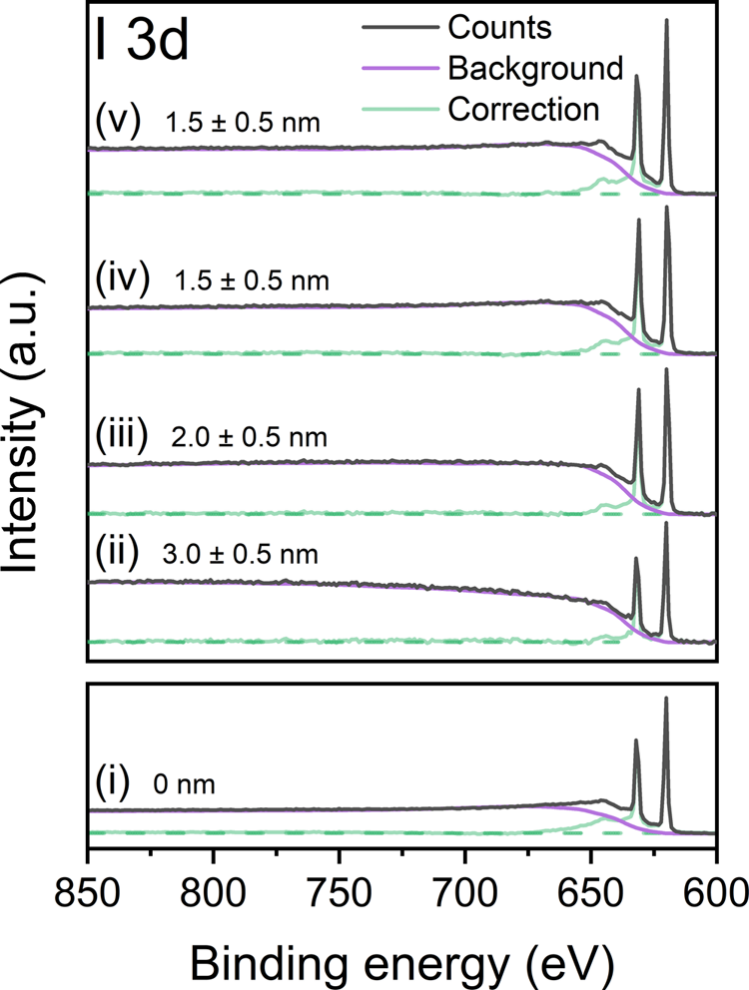
Inelastic background fits to the I 3d region of XPS survey scans of the (i) 0 wt % film at RT, and the 5 wt % film at (ii) RT, (iii) 100 °C, (iv) 150 °C, and (v) 180 °C, generated using the QUASES-Tougaard software.^58,59^ The purple line corresponds to a simulated inelastic background from MAPI topped with various pHEMA overlayer thicknesses (displayed), and the black line shows the true background.

The existence of a pHEMA overlayer is also suggested by the XPS survey spectra from pristine 0 and 5 wt % films, shown in Figure S7. The 5 wt % inelastic background displays shoulders to the higher BE side of strong core level peaks, characteristic of the attenuation of photoelectrons as they pass through an overlayer. It is possible that excess pHEMA at the surface of the 5 wt % film causes photoelectrons to lose energy before reaching the detector, resulting in higher recorded BEs. The QUASES-Tougaard inelastic background modeling software was used to determine the “effective” thickness of any potential pHEMA overlayer, assuming a 100% coverage of polymer.^58,59^ While the system is unlikely to be segregated into layers to such a degree, the calculation yields a useful measure of the excess of pHEMA at the surface. The IMFP of I 3d photoelectrons (867 eV KE) through pHEMA, calculated as 3 nm using the TPP-2M formula, was included in the overlayer model.^60,61^ The thickness of the pHEMA overlayer was then optimized by fitting to the inelastic background following the I 3d peak in the XPS survey spectra. Figure 6 illustrates the model fit to the 0 wt % survey spectra at RT (to confirm the absence of an overlayer) and 5 wt % survey spectra at all temperatures, suggesting that an initial effective pHEMA coverage of 3.0 ± 0.5 nm at RT reduces to 1.5 ± 0.5 nm at 180 °C.

Evidence of loss of pHEMA at high temperature can be seen in the C 1s and O 1s high-resolution core-level spectra, shown in Figure S8. A reduction in intensity of both peaks can be seen as temperature increases. Ratios of the intensity of the C 1s peak at 289.7 eV BE (labeled C_O–C=O_ in Figure S8), attributed to the O–C=O bonding environment in pHEMA, and Pb^2+^ were calculated and are displayed in Figure S9. The reduction of C_O–C=O_/Pb^2+^ mirrors the reduction in overlayer thickness shown in Figure 6, reinforcing the conclusion that this layer is composed primarily of pHEMA.

The presence of a pHEMA overlayer offers an explanation for the reduced I/Pb^2+^ ratio calculated for the 5 wt % film. Pb 4f photoelectrons possess a higher KE than those from the I 3d core level. As a result, the Pb 4f signal is more representative of the bulk (IMFPs I 3d_5/2_ = 3 nm, Pb 4f_7/2_ = 4 nm), whereas the signal from the I 3d core level is more surface sensitive. Thus, a reduction in I intensity relative to Pb should be expected if the surface is depleted in I (and Pb) due to the presence of a pHEMA overlayer. Estimated observed stoichiometric ratios for MAPI, in the presence of a pHEMA-rich surface layer with an estimated thickness of 3 nm, are calculated as 1:1.5:0.7 (Pb^2+^:I:N, see Table S6). These adjusted values better reflect the 5 wt % ratios obtained at RT. The influence of the overlayer on the calculated elemental concentration will be reduced as the temperature is raised, due to the loss of pHEMA at higher temperatures.

### 3.7 Thermal Decomposition at 9 mbar Water Vapor Pressure

**Figure 7:**
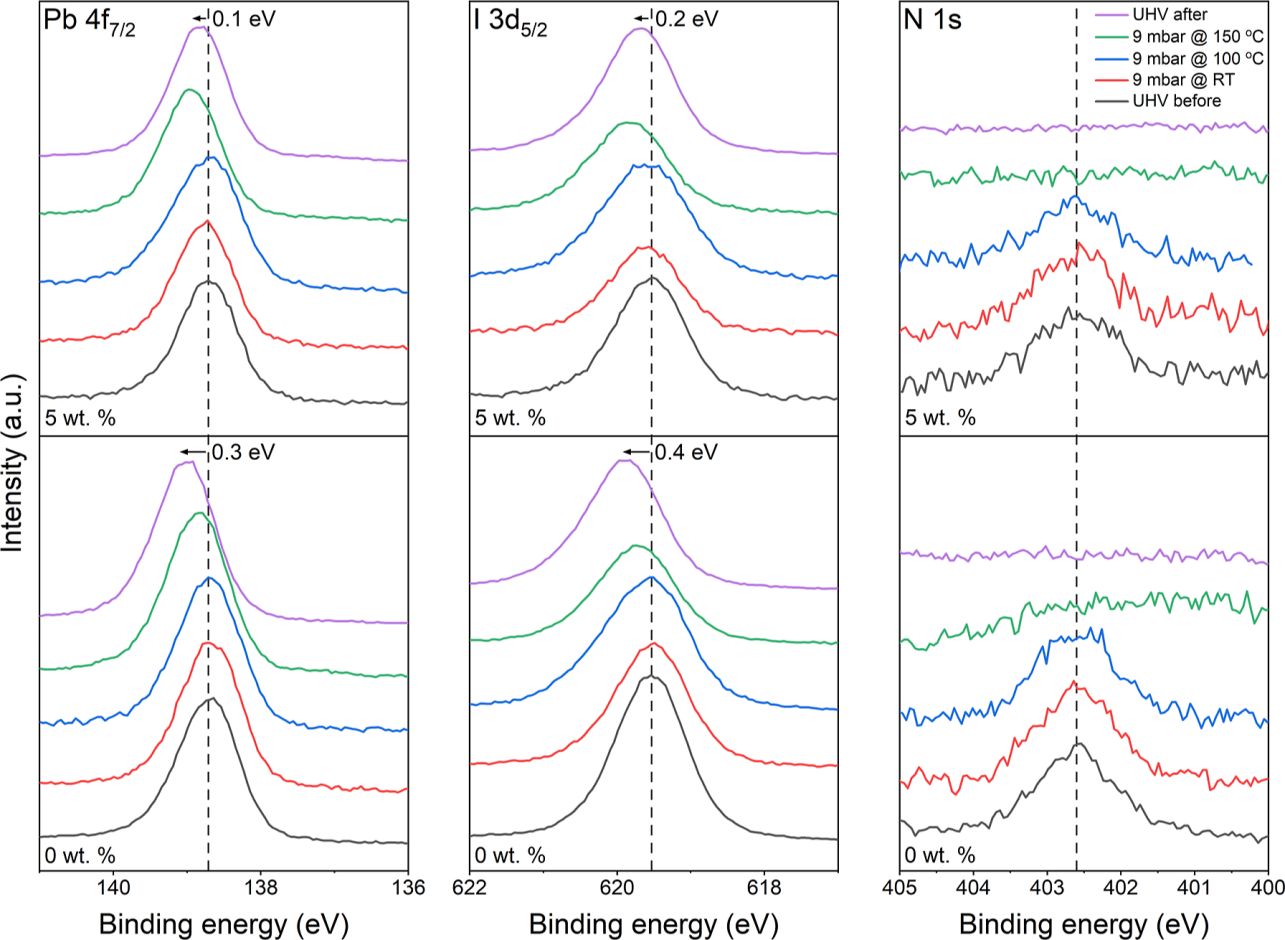
Pb 4f_7/2_, I 3d_5/2_, and N 1s (left to right) high-resolution core-level NAP-XPS spectra of the 0 wt % (bottom) and 5 wt % (top) films. Measurements were taken at RT under UHV conditions (before), RT with 9 mbar water vapor pressure, 100 °C with 9 mbar, 150 °C with 9 mbar, and again at RT under UHV conditions (after). Initial peak positions are indicated by the dashed lines, and the final shift is noted (as measured after exposure).

To further assess the role of water, we employed NAP-XPS to follow a similar heating regime while simultaneously exposing 0 and 5 wt % films to 9 mbar water vapor pressure (∼30% RH). This was the maximum humidity achievable in this in situ experiment while maintaining viable count rates. Measurements were taken at RT under UHV conditions (before exposure), then at RT, 100, and 150 °C under sustained water vapor exposure, and finally at RT under UHV conditions (after exposure), following the removal of water vapor via pumping. Pb 4f_7/2_, I 3d_5/2_, and N 1s high-resolution core-level NAP-XPS spectra are displayed in Figure 7.

Initially, the Pb^2+^ feature is observed at 138.7 ± 0.1 eV BE for both samples, consistent with our XPS study. A small peak at 137.0 ± 0.1 eV is present in both spectra following exposure, corresponding to Pb^0^. Following exposure, a shift in BE of +0.3 eV is observed in the 0 wt % Pb 4f_7/2_ spectrum, while the 5 wt % spectrum displays a +0.3 eV shift at 150 °C and reduces to only +0.1 eV following the removal of water vapor. A similar displacement is observed in the I 3d spectra, in which the peaks shift from an initial position of 619.5 ± 0.1 eV by +0.4 and +0.2 eV BE in the 0 and 5 wt % spectra, respectively. Again, the maximum shift exhibited by the 5 wt % film is +0.3 eV at 150 °C. As observed in our UHV study, the MA^+^ peak in the N 1s spectra at 402.6 ± 0.1 eV BE disappears completely following heating.

Elemental concentrations were calculated before and after exposure to water vapor from peaks fitted to the high-resolution core-level NAP-XPS spectra. The initial I/Pb^2+^ ratio for the 0 wt % film was found to be 2.1 ± 0.1, and this reduces to 1.5 ± 0.1 following exposure. As observed in our UHV study, the concentration of iodine is less in the 5 wt % sample, likely due to the presence of a pHEMA overlayer as discussed previously. This ratio was found to be 1.4 ± 0.1 and reduces only marginally to 1.3 ± 0.1 following exposure. The discrepancy between the pristine I/Pb^2+^ ratios calculated here and during our UHV study is likely due to sample degradation between fabrication and analysis, as both samples were stored under the same conditions prior to measurement. The N/Pb^2+^ ratios for the 0 and 5 wt % films were found to be 0.6 ± 0.1 and 0.7 ± 0.1, respectively. Following exposure to water and heating, the nitrogen concentration reduced to zero in both cases. A small concentration of Pb^0^ was evident following exposure in both the 0 and 5 wt % films, with Pb^0^/Pb^2+^ ratios of 0.01 ± 0.01 and 0.03 ± 0.01 respectively.

The more prominent shift in Pb 4f_7/2_ and I 3d_5/2_ peak BEs observed in the 0 wt % spectra (i.e., no added pHEMA), as well as the greater change in the I/Pb^2+^ ratio, suggests moisture-induced degradation of MAPI to PbI_2_ occurs with a subsequent loss of iodine in the form of HI gas (in accordance with the process outlined by eq 1). Inclusion of 5 wt % pHEMA in the perovskite film leads to smaller BE shifts following the removal of water vapor, which may be an indication of “self-healing” of the MAPI, discussed further in Section 4.^35,43,44^

To provide further insights into the effect of pHEMA on the stability of MAPI, XPS was performed on MAPI containing various concentrations of pHEMA (0–10 wt %), aged ex situ in 35 and 70% RH, matching the controlled humidities used for the bulk characterization analyses (see Section 3.3). Figures S10–S12 display high-resolution core-level spectra and elemental concentrations from these samples. We cannot interpret BE shifts in core-level peaks as evidence of increased degradation, as the same measurement position could not be maintained due to ex situ aging. However, compositional analysis does reveal that Pb^0^ increases more rapidly in pure MAPI compared to all concentrations of pHEMA studied, as observed in our in situ degradation studies.

### 3.8 Angle-Resolved HAXPES Depth Profiling

Angle-resolved HAXPES was used to provide a depth profile of the 0 and 5 wt % films. High-resolution spectra of the Pb 3d_5/2_ and I 2p_3/2_ core levels (2483 and 4556 eV BE, respectively) are displayed in Figure S13. The spectra display a reduction in the I concentration with increased electron emission angle. The electron emission angle was changed to affect the sampling depth of the instrument, according to eq 3, with higher electron emission angle relative to the surface normal corresponding to a lower sampling depth. The TPP-2M formula was used to determine the IMFP of photoelectrons from each core level.^60,61^ These values were then input into eq 3 to determine the approximate sampling depth for each electron emission angle. The IMFPs of photoelectrons through pHEMA were found to be similar to those calculated for MAPI; thus, the effect of any possible pHEMA overlayer is neglected.

**Figure 8:**
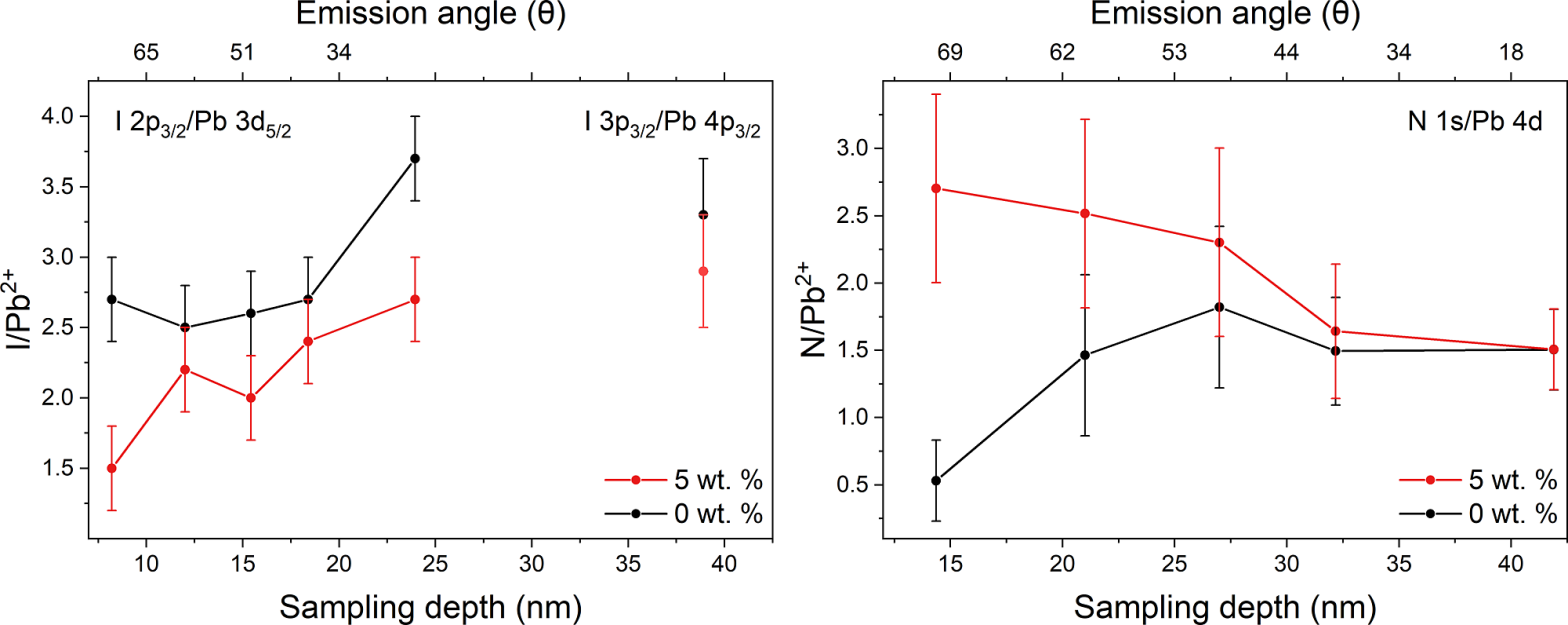
I/Pb^2+^ and N/Pb^2+^ elemental ratios calculated from peaks fitted to the high-resolution core level HAXPES data, as a function of the estimated sampling depth. Sampling depth was calculated using eq 3, and is approximated using the IMFP of the numerator signal in all cases (IMFPs I 2p_3/2_ = 8 nm, I 3p_3/2_ = 13 nm, and N 1s = 14 nm). Electron emission angle relative to the surface normal is displayed on the top axis. An I 2p_3/2_/Pb 3d_5/2_ error of 0.3 nm was maintained by increasing the integration time to negate the drop in signal at large angles. Values are shown in Tables S7 and S8.

Figure 8 displays the I/Pb^2+^ and N/Pb^2+^ ratios for each sample as a function of the estimated sampling depth, approximated using the IMFP of the numerator signal. The I/Pb^2+^ ratios at an estimated depth of 24 nm were found to be 3.7 ± 0.2 and 2.7 ± 0.3 for the 0 and 5 wt % films, respectively, close to the nominal value of 3.0. A reduction in I/Pb^2+^ at the surface is exhibited by both samples, 2.7 ± 0.3 and 1.5 ± 0.2 at 8 nm for the 0 and 5 wt % films, respectively; however, the reduction is larger in the 5 wt % film. An additional concentration, calculated as the ratio of I 3p_3/2_ (873 eV BE) and Pb 4p_3/2_ (643 eV BE) peak intensities at θ = 4°, is included in Figure 8. Due to much larger photoelectron KEs, these core levels provide a more bulk sensitive elemental ratio. The sampling depth is approximated to that of I 3p_3/2_ photoelectrons, found to be 39 nm. The I/Pb^2+^ ratios at this depth were found to be 3.3 ± 0.4 and 2.9 ± 0.4 for the 0 and 5 wt % films, respectively, close to the nominal value for MAPI. It should be noted that at larger emission angles, surface roughness is often a limiting factor due to shadowing effects; thus, quantification obtained by conventional XPS (sampling depth of I 3d_5/2_ = 6 nm) is believed to be more representative of the surface stoichiometry.^75,76^

The N/Pb^2+^ ratios at an estimated sampling depth of 42 nm were found to be 1.5 ± 0.3 for both the 0 and 5 wt % films, close to the nominal value of 1.0. This ratio reduces with sampling depth in the 0 wt % film to 0.5 ± 0.3 at 14 nm, likely to be due to degradation of the organic cation at the surface of the film caused by atmospheric exposure. In marked contrast, the N/Pb^2+^ ratio increases at the surface of the 5 wt % film, to a maximum value of 2.7 ± 0.7 at 14 nm estimated sampling depth. This increase may be an indication that the pHEMA overlayer is able to “trap” N-containing moieties produced due to atmospheric exposure of MAPI. We note here that samples were stored in an evacuated desiccator for one week prior to HAXPES analysis, rather than in a nitrogen-filled glovebox, as was the case in our XPS and NAP-XPS studies. As a result, we expect to see evidence of greater degradation in our HAXPES quantification than in our XPS and NAP-XPS measurements from fresh samples. The high N/Pb^2+^ ratio at low sampling depths found in HAXPES of the 5 wt % film is in contrast with that obtained from XPS (shown in Figure 5, sampling depth of N 1s = 8 nm), providing evidence that when the films degrade, N-containing degradation products remain trapped in the pHEMA overlayer, rather than leaving the surface completely, as is seen in the 0 wt % film.

Inelastic background modeling was used to determine the thickness of any possible pHEMA overlayer on the 5 wt % film, using the I 2p_3/2_ and Pb 3p_3/2_ regions of the HAXPES survey.^58^ The IMFPs of photoelectrons from these core levels were calculated using the TPP-2M formula as 10 and 13 nm, respectively.^60,61^ An inelastic background model based on these two values and an overlayer thickness of 3.0 ± 0.5 nm fitted the data well, and is in excellent agreement with the thickness estimated previously using the I 3d high-resolution core-level XPS spectrum. The inelastic background produced by this overlayer model is displayed in Figure S14.

The observations (from both XPS and HAXPES) of a high concentration of pHEMA at the surface of this perovskite sample means that we must be cautious in interpreting the elemental ratios obtained from this non-uniform sample. As in the XPS measurements, in our HAXPES experiments, the Pb signal used in obtaining the elemental I/Pb^2+^ ratio has a significantly higher KE and, thus, sampling depth than the corresponding I feature. This means that the I signal is more attenuated by the pHEMA overlayer than the Pb signal and leads to an underestimation of the I/Pb^2+^ ratios in the 5 wt % film. This effect is exaggerated at high emission angles, as photoelectrons travel through more of the overlayer, contributing to the low values observed in HAXPES (see Figure 8). However, the effect of the pHEMA overlayer on the N/Pb^2+^ ratios obtained from the HAXPES measurements should not be significant, due to the small KE separation of the Pb 4d_5/2_ and N 1s core levels (∼10 eV, cf. > 2000 eV for I 2p_3/2_ and Pb 3d_5/2_ core levels). The influence of the pHEMA overlayer on each pair of core levels is indicated by the corrected nominal stoichiometric ratios presented in Table S6.

## 4 Discussion

It has been demonstrated previously that on exposure to water vapor, MAPI adopts two intermediate hydrated phases before degrading irreversibly via the process displayed in eq 1.^16,43^
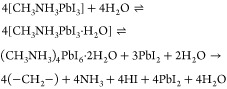
4

The monohydrate phase (CH_3_NH_3_PbI_2_·H_2_O) readily converts back to MAPI on dehydration.^77^ The reversibility from the dihydrate phase [(CH_3_NH_3_)_4_PbI_6_·2H_2_O], formed following prolonged exposure to water vapor, is limited by the phase separation of the reaction products. In spite of this, the hydration process outlined in eq 4 has been shown to be fully reversible following exposure to moderate humidity in the absence of condensed water.^43^

**Figure 9:**
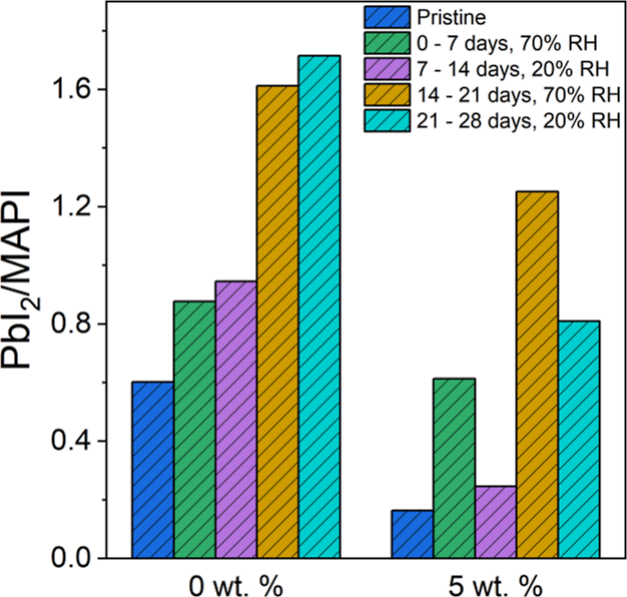
PbI_2_(001)/MAPI(110) peak intensity ratio of the various aging perovskite films kept in varying humidity (pristine, 0–7 days at 70% RH, 7–14 days at 20% RH, 14–21 days at 70% RH, and 21–28 days at 20% RH).

To test the influence of pHEMA additives on this “self-healing” mechanism, we simulated two specific humidity conditions in the laboratory: storage in a dry box containing water-absorbing gel (20% RH) and exposure to a constant moisture environment box (70% RH), using the methodology described in the Experimental Section. At 7 day intervals, MAPI films were alternately placed in low and very high humidity environments, and their compositional changes were monitored by XRD. Stability was inferred from the PbI_2_(100)/MAPI(110) reflection intensity ratios extracted from the XRD patterns (Figure S15), displayed in Figure 9.

After seven days of aging in an environment with 70% RH, the intensity ratio of the PbI_2_/MAPI reflections for 0 wt % MAPI increased significantly, indicating that the MAPI film without the support of the cross-linked hydrogel network readily decomposes into the PbI_2_ phase. The sample was then stored in a 20% RH environment for seven days, during which time the intensity ratio of the PbI_2_/MAPI reflections increased slightly. The film was then placed at 70% RH for seven days, whereupon the intensity ratio of the PbI_2_/MAPI reflections increased sharply. Finally, the sample was again stored in 20% RH for seven days, after which the peak intensity ratio of PbI_2_/MAPI reflections displayed a slight increase. A marked difference was displayed by 5 wt % pHEMA-MAPI samples. After seven days of aging in 70% RH, the intensity ratio of the PbI_2_/MAPI reflections also increased significantly, but after storage at 20% RH for seven days, the intensity ratio of the PbI_2_/MAPI reflections returned to close to the value for the fresh state, suggesting that the inclusion of pHEMA enhanced the back reaction (eq 4), involving conversion of PbI_2_ back to MAPI.^35,43,44^ When placed in 70% RH for 7 days, the peak intensity ratio of PbI_2_/MAPI began to increase sharply as before. Finally, the sample was stored in 20% RH for 7 days, and the intensity ratio of the PbI_2_/MAPI reflections again decreased significantly. We propose that the inclusion of pHEMA enhances the self-healing process by both reducing the contact of water vapor with MAPI crystals and preventing the release of decomposition products, allowing MAPI to re-form following a reduction in humidity. The ability of pHEMA to absorb moisture from the environment reduces the exposure of MAPI grains to water vapor, which consequently reduces the decomposition of the pHEMA-incorporated MAPI film beyond the dihydrate phase.^74^ Following a reduction in humidity, these hydrated phases convert back to MAPI following the process outlined in eq 4, resulting in a larger self-healing effect in pHEMA-incorporated MAPI films.^43^

In a previous study of PEG-incorporated MAPI films by Zhao et al., it was reported that hydrogen bonding between the polymer and MAI (evidenced by NMR measurements) restricted the loss of nitrogen and iodine from the surface following exposure to water vapor.^35^ The captured decomposition products subsequently reacted to re-form MAPI following the removal of water vapor. It is possible that this effect is also exhibited by pHEMA, contributing to the self-healing process. The increased nitrogen concentration at the surface of the pHEMA-incorporated film, compared with pure MAPI, calculated from HAXPES spectra and displayed in Figure 8, supports this conclusion. While nitrogen in the form of ammonia gas is lost from the surface of pure MAPI due to atmospheric exposure following the process outlined in eq 1, this decomposition product is trapped by the polymer in pHEMA-incorporated MAPI. As evidenced by inelastic background analysis (see Figure 6), a large concentration of pHEMA is present at the surface of the film; thus, an increased concentration of trapped nitrogen degradation products is expected at the surface of pHEMA-incorporated MAPI, in line with experimental observations. Interestingly, we do not observe a similar increase in the I concentration at the surface, which may suggest that I-containing degradation products are not trapped by the pHEMA overlayer. However, as mentioned in Section 3.8, due to differential attenuation by the overlayer, the 5 wt % I/Pb^2+^ ratios calculated using HAXPES are expected to be significantly underestimated and are more in line with the corrected nominal stoichiometric values displayed in Table S6.

**Figure 10:**
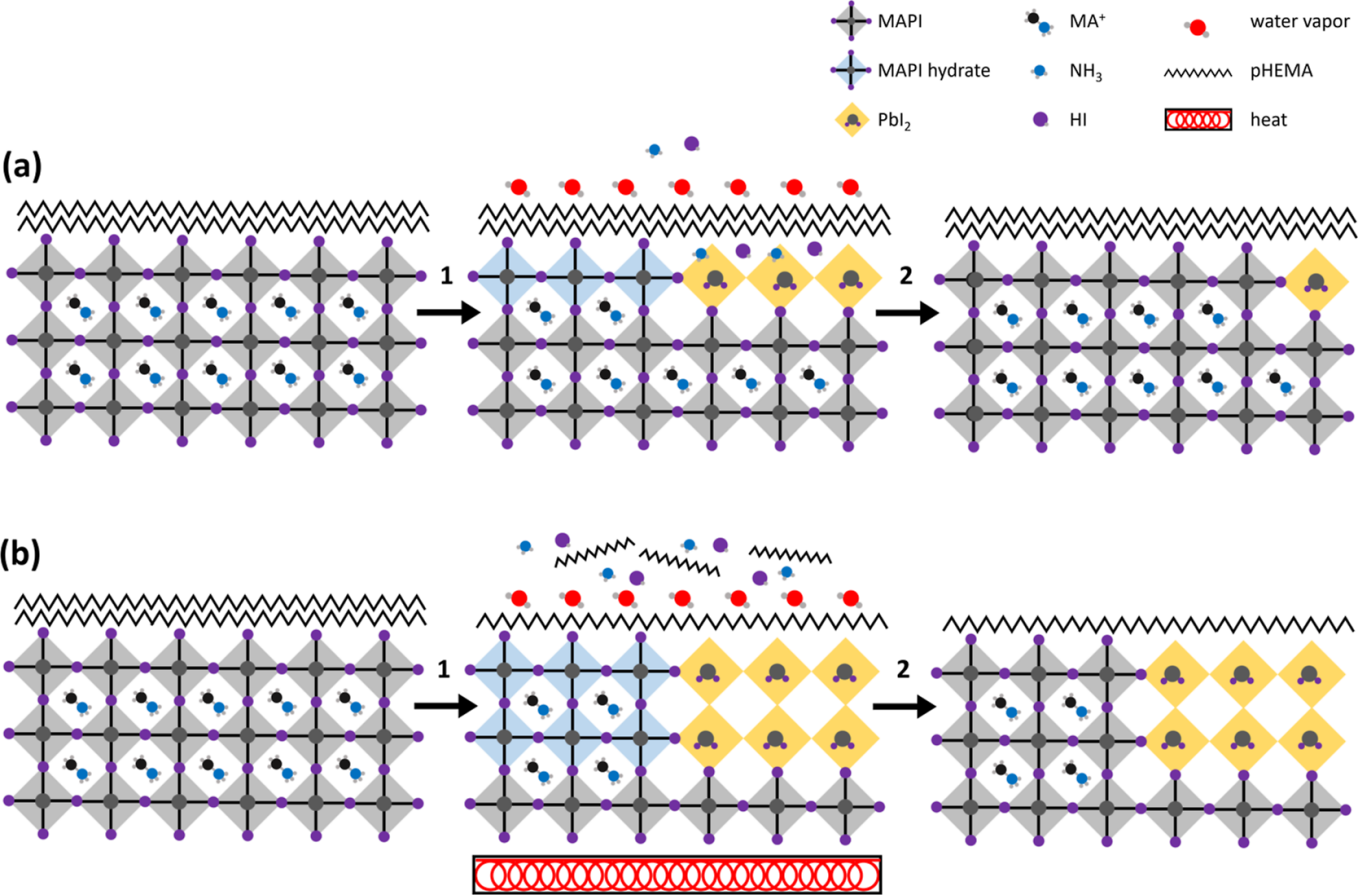
Schematic diagram of pHEMA-incorporated MAPI films when exposed to water (a) and water and heat (b). Steps 1 and 2 represent the introduction and removal of these conditions. Organic residue from MA^+^ is not included in this diagram.

Evidence of pHEMA loss at high temperatures, also revealed through inelastic background analysis, provides an explanation regarding why this self-healing process is not as apparent in our NAP-XPS data, where the surface was heated in UHV. The loss of pHEMA from the surface of the pHEMA-incorporated MAPI film at high temperatures, shown in Figures 6, S8 and S9, results in both reduced moisture protection and reduced retention of decomposition products. As a result, the self-healing process demonstrated at RT by RD in Figure 9 is seriously diminished following heating to 150 °C. A schematic of these processes at both RT and on heating is displayed in Figure 10.

## 5 Conclusions

We have investigated the surface degradation of MAPI films with pHEMA as an additive, as a function of temperature, water, and vapor pressure and have also monitored the effect of ambient air exposure on the performance of functioning solar cells based on this material. The inclusion of pHEMA as an additive in the precursor solution is shown to produce high-quality MAPI films with a larger grain size and a reduced concentration of PbI_2_ compared to those prepared via a conventional two-step route. This improvement can be attributed to the formation of porous PbI_2_ caused by the escape of the degradation products of pHEMA during the annealing process, allowing for easier incorporation of MAI into the material in the second step of the synthesis and thus greater conversion of PbI_2_ to MAPI following the addition of MAI. The HEMA molecule is demonstrated to be deposited intact from NEXAFS measurements. Devices based on pHEMA-incorporated MAPI exhibited enhanced cell characteristics. A champion PCE of 17.8% was obtained from a 5 wt % device, compared to 16.5% for 0 wt %. The 5 wt % pHEMA-MAPI device is shown to retain 90.2% of its best efficiency after 500 h at 70% RH, in contrast with the 64.2% achieved using a pure MAPI device. XPS shows that pHEMA-incorporated MAPI films exhibit less degradation than pure MAPI on annealing and in the presence of water, with evidence of reduced degradation to both PbI_2_ and Pb^0^. Strong evidence for the passivation of grain boundaries by pHEMA is provided by surface stoichiometry values from XPS and inelastic background measurements, which reveal the presence of a pHEMA-rich layer at the surface with an effective thickness of ca. 3 nm. Enhanced reversible degradation of the pHEMA-incorporated MAPI film via a self-healing process is demonstrated by XRD. In addition, non-destructive, angle-resolved HAXPES reveals an increased surface N/Pb^2+^ ratio, consistent with the hypothesis that degradation products produced due to high humidity are trapped by the pHEMA overlayer and can be reincorporated into the perovskite following dehydration. Our measurements illustrate the benefits of hydrophilic polymer additives such as pHEMA and shed light on the mechanisms behind the enhanced stability and self-healing of such perovskite composites.

## Supporting Information Available

PEY NEXAFS of C K-edge and N K-edge of 0 and 5 wt % samples, and DFT simulated HEMA C K-edge; UV–vis–NIR absorbance spectra, PL spectra, and XRD patterns of 0–10 wt % films; photovoltaic parameters of 0–10 wt % devices, pristine and during storage at 35 and 70% RH; XRD patterns and UV–vis–NIR absorbance spectra of 0–10 wt % films during storage at 35 and 70% RH; images of films during storage at 35% and 70% RH; tabulated XPS elemental ratios of 0 and 5 wt % films during heating at UHV; C 1s and O 1s core-level XPS spectra and survey spectra of pristine 0 and 5 wt % films; C 1s and O 1s core-level XPS spectra of 5 wt % film during heating at UHV; C_O–C=O_ elemental ratio as a function of temperature in 5 wt % film; nominal stoichiometric ratio overlayer correction and tabulated values, Pb 4f_7/2_, I 3d_5/2_, and N 1s core-level XPS spectra of 0–10 wt % films at various stages of aging ex situ at 35 and 70% and the corresponding elemental ratios; Pb 3d_5/2_, and I 2p_3/2_ core-level HAXPES spectra as a function of electron emission angle; tabulated HAXPES elemental ratios as a function of estimated sampling depth; QUASES inelastic background fitting to I 2p_3/2_ and Pb 3p_3/2_ HAXPES peaks from 5 wt % film; and XRD patterns of 0–10 wt % films alternated between 20 and 70% RH storage (PDF)

## Notes

The data associated with this work are openly available, see DOI: 10.48420/21750464.

## Abbreviations

OHP: organometal halide perovskite
pHEMA: poly(2-hydroxyethyl methacrylate)
MAPI: methylammonium lead iodide perovskite (CH_3_NH_3_PbI_3_)
PCE: power conversion efficiency
XRD: X-ray diffraction
XPS: X-ray photoelectron spectroscopy
NAP-XPS: near-ambient pressure XPS
HAXPES: hard XPS
NEXAFS: near-edge X-ray absorption fine structure
PSC: perovskite solar cell
PEG: polyethylene glycol
MAI: methylammonium iodide (CH_3_NH_3_I)
ITO: indium tin oxide
HTL: hole transport layer
RT: room temperature
UV–vis–NIR: ultraviolet–visible–near-infrared
RH: relative humidity
UHV: ultra-high vacuum
BE: binding energy
RSF: relative sensitivity factor
KE: kinetic energy
IMFP: inelastic mean free path
PEY: partial electron yield
fwhm: full width half maximum
TPP-2M: Tanuma–Powell–Penn formula
SEM: scanning electron microscopy
*J*_SC_: short circuit current density
*V*_OC_: open circuit voltage
FF: fill-factor
MA^+^: methylammonium cation
