# Supplementary material for: Elucidating the Mechanism of Self-Healing in Hydrogel-Lead Halide Perovskite Composites for Use in Photovoltaic Devices
Source: ACS Appl Mater Interfaces. 2023 May 30;15(23):28008–22. doi: 10.1021/acsami.3c03359 (PMC10273229; doi:10.1021/acsami.3c03359)
Supplement: Supplementary file 1 — am3c03359_si_001.pdf [file am3c03359_si_001.pdf]

SUPPORTING INFORMATION

Elucidating the mechanism of self-healing in hydrogel-lead halide perovskite composites for use in photovoltaic devices

Dawei Zhao,[ab‡] Tom A. Flavell,[ac‡] Fahad Aljuaid,[ab] Stephen Edmondson,[b] Ben F. Spencer,[ad] Alex S. Walton,[ae] Andrew G. Thomas[ab] and Wendy R. Flavell[ac]

a. Photon Science Institute, University of Manchester, Oxford Road, Manchester, M13 9PL, UK.

b. Department of Materials, University of Manchester, Oxford Road, Manchester, M13 9PL, UK.

c. Department of Physics and Astronomy, University of Manchester, Oxford Road, Manchester, M13 9PL, UK.

d. Henry Royce Institute, University of Manchester, Oxford Road, Manchester, M13 9PL, UK.

e. Department of Chemistry, University of Manchester, Oxford Road, Manchester, M13 9PL, UK.

‡ These authors contributed equally

NEXAFS

NEXAFS was performed on all samples to identify resonant electronic transitions from core states to antibonding molecular orbitals. Carbon and nitrogen K-edge NEXAFS spectra from the fresh MAPI and MAPI-pHEMA films are displayed in Figure S1. Both total electron yield (TEY) and partial electron yield (PEY, 270 V cut-off) are displayed, providing more bulk and surface sensitive spectra respectively. The peaks at 287.5 eV and 290 eV in the carbon K-edge spectrum of pure MAPI are associated with the C-H and C-N σ* bonds present in the perovskite MA^+ cation respectively.[1] Also present in the pure MAPI spectrum is a broad feature centred at 284.2 eV (labelled C1), an energy usually associated with π* orbitals not found in perovskite materials. Previous reports have associated this feature with beam damage and adventitious carbon.[1] The carbon K-edge spectra from the MAPI-pHEMA film contain an additional strong peak at 288.4 eV originating from the C=O π* transition within the pHEMA.[2] For comparison the carbon K-edge NEXAFS spectrum for pHEMA was simulated using DFT performed in the StoBe-deMon software.[3] This spectrum, displayed in green in Figure S1, also exhibits a single C=O π* feature, consistent with experimental observations.

As expected, the nitrogen K-edge spectra from both samples contain the same features. The main edge at 405.4 eV is assigned as convolution of both N-C σ* and N-H σ* orbitals present in the perovskite MA^+ cation.[4] The small peak at 400 eV (labelled N1) is assumed to be due to minor contaminations on the sample surface.

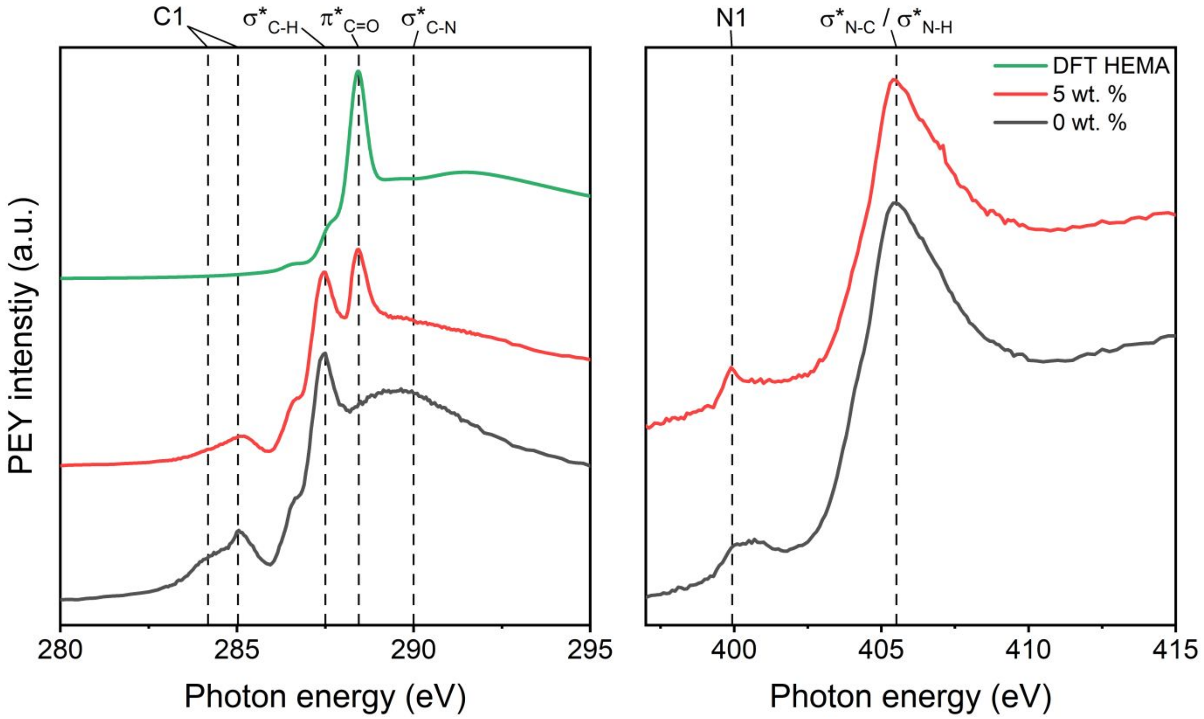

Figure S1. Carbon K-edge (left) and nitrogen K-edge (right) PEY NEXAFS spectra from 0 wt. % and 5 wt. % films with prominent features are labelled. A DFT simulated HEMA carbon K-edge spectrum is included for comparison.

Morphology, device structure and performance

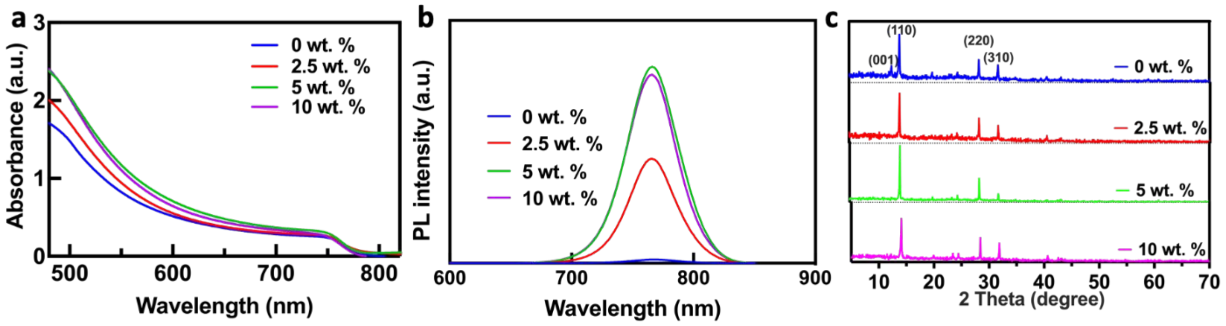

Figure S2. (a) UV-VIS-NIR absorbance spectra, (b) PL spectra, (c) and XRD patterns of the perovskite thin films with different concentrations of pHEMA.

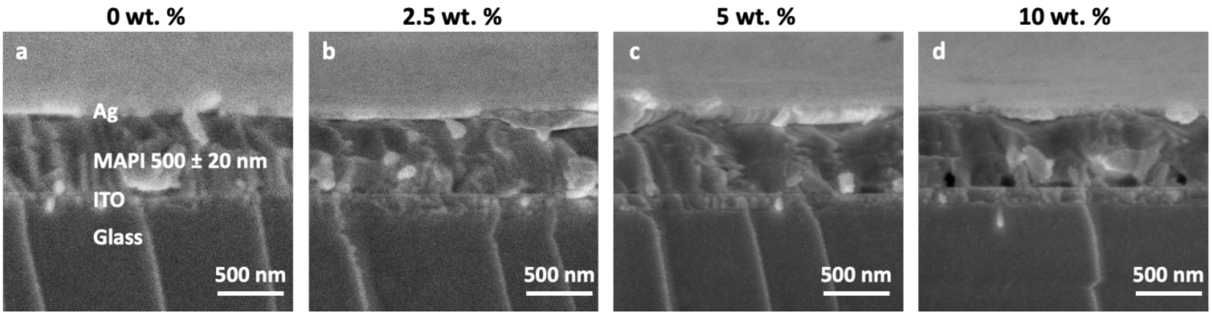

Figure S3. Cross-sectional SEM images of MAPI films with various concentrations of pHEMA (0 – 10 wt. %).

**Samples**	**Scan direction**	J_{SC} **(mA/cm²)**	V_{OC} **(V)**	**FF (%)**	**PCE (%)**
**0 wt. %**	R	22.3 ± 0.1	1.02 ± 0.02	72.5 ± 0.3	16.4 ± 0.2
**2.5 wt. %**	R	22.5 ± 0.2	1.04 ± 0.02	73.8 ± 0.8	17.2 ± 0.1
**5 wt. %**	R	22.6 ± 0.2	1.03 ± 0.02	76.3 ± 0.8	17.8 ± 0.1
**10 wt. %**	R	22.5 ± 0.2	1.04 ± 0.02	73.7 ± 0.8	17.3 ± 0.1

R=Reverse scan direction

Table S1. The photovoltaic parameters of PSCs with different concentration of pHEMA.

Evolution of photovoltaic performance in humid environments

Samples	Parameters	0 day	1 day	4 day	8 day	12 day	16 day	20 day	30 day	40 day	50 day	60 day
**0 wt. %**	**PCE (%)**	15.1 ± 0.2	15.7 ± 0.2	16.4 ± 0.2	16.5 ± 0.2	15.8 ± 0.2	14.6 ± 0.2	14.1 ± 0.2	13.4 ± 0.2	12.7 ± 0.2	11.5 ± 0.2	11.3 ± 0.2
	**J_{sc} (mA/cm^2)**	22.2 ± 0.1	22.3 ± 0.1	22.3 ± 0.1	22.2 ± 0.1	22.3 ± 0.1	21.7 ± 0.1	21.7 ± 0.1	21.6 ± 0.1	21.5 ± 0.1	21.7 ± 0.1	21.7 ± 0.1
	**V_{OC} (V)**	1.03 ± 0.02	1.03 ± 0.02	1.03 ± 0.02	1.03 ± 0.02	1.03 ± 0.02	1.02 ± 0.02	1.02 ± 0.02	1.02 ± 0.02	1.02 ± 0.02	1.01 ± 0.02	1.01 ± 0.02
	**FF (%)**	66.1 ± 0.3	68.7 ± 0.3	71.5 ± 0.3	72.3 ± 0.3	69.0 ± 0.3	66.2 ± 0.3	64.1 ± 0.3	60.9 ± 0.3	57.9 ± 0.3	52.6 ± 0.3	51.8 ± 0.3
**5 wt. %**	**PCE (%)**	16.6 ± 0.1	16.9 ± 0.1	17.4 ± 0.1	17.8 ± 0.1	17.6 ± 0.1	17.3 ± 0.1	17.3 ± 0.1	17.2 ± 0.1	17.2 ± 0.1	17.1 ± 0.1	17.0 ± 0.1
	**J_{sc} (mA/cm^2)**	22.6 ± 0.2	22.6 ± 0.2	22.6 ± 0.2	22.6 ± 0.2	22.4 ± 0.2	22.4 ± 0.2	22.2 ± 0.2	22.0 ± 0.2	21.7 ± 0.2	21.7 ± 0.2	21.9 ± 0.2
	**V_{OC} (V)**	1.03 ± 0.02	1.04 ± 0.02	1.04 ± 0.02	1.04 ± 0.02	1.03 ± 0.02	1.03 ± 0.02	1.01 ± 0.02	1.01 ± 0.02	1.02 ± 0.02	1.03 ± 0.02	1.04 ± 0.02
	**FF (%)**	70.9 ± 0.8	72.3 ± 0.8	74.1 ± 0.8	75.7 ± 0.8	76.1 ± 0.8	75.0 ± 0.8	76.9 ± 0.8	77.3 ± 0.8	77.5 ± 0.8	76.5 ± 0.8	75.0 ± 0.8

Table S2 The photovoltaic parameters of devices aged under 35 % RH.

Samples	Parameters	0 day	1 day	4 day	8 day	12 day	16 day	20 day
**0 wt. %**	**PCE (%)**	15.0 ± 0.2	16.3 ± 0.2	16.5 ± 0.2	14.5 ± 0.2	12.8 ± 0.2	11.3 ± 0.2	10.6 ± 0.2
	**J_{sc} (mA/cm^2)**	22.2 ± 0.1	22.2 ± 0.1	22.2 ± 0.1	21.8 ± 0.1	21.7 ± 0.1	19.1 ± 0.1	19.0 ± 0.1
	**V_{OC} (V)**	1.02 ± 0.02	1.02 ± 0.02	1.02 ± 0.02	1.01 ± 0.02	1.01 ± 0.02	1.02 ± 0.02	1.01 ± 0.02
	**FF (%)**	66.1 ± 0.3	71.7 ± 0.3	73.1 ± 0.3	65.9 ± 0.3	58.4 ± 0.3	58.3 ± 0.3	55.1 ± 0.3
**5 wt. %**	**PCE (%)**	16.7 ± 0.1	17.3 ± 0.1	17.8 ± 0.1	17.4 ± 0.1	17.1 ± 0.1	16.4 ± 0.1	16.1 ± 0.1
	**J_{sc} (mA/cm^2)**	22.5 ± 0.2	22.4 ± 0.2	22.3 ± 0.2	22.2 ± 0.2	22.0 ± 0.2	21.5 ± 0.2	21.2 ± 0.2
	**V_{OC} (V)**	1.03 ± 0.02	1.03 ± 0.02	1.04 ± 0.02	1.04 ± 0.02	1.03 ± 0.02	1.02 ± 0.02	1.04 ± 0.02
	**FF (%)**	72.6 ± 0.8	74.7 ± 0.8	76.7 ± 0.8	75.3 ± 0.8	75.5 ± 0.8	74.2 ± 0.8	73.1 ± 0.8

Table S3. The photovoltaic parameters of devices aged under 70 % RH.

XRD patterns and UV-VIS-NIR spectra during degradation

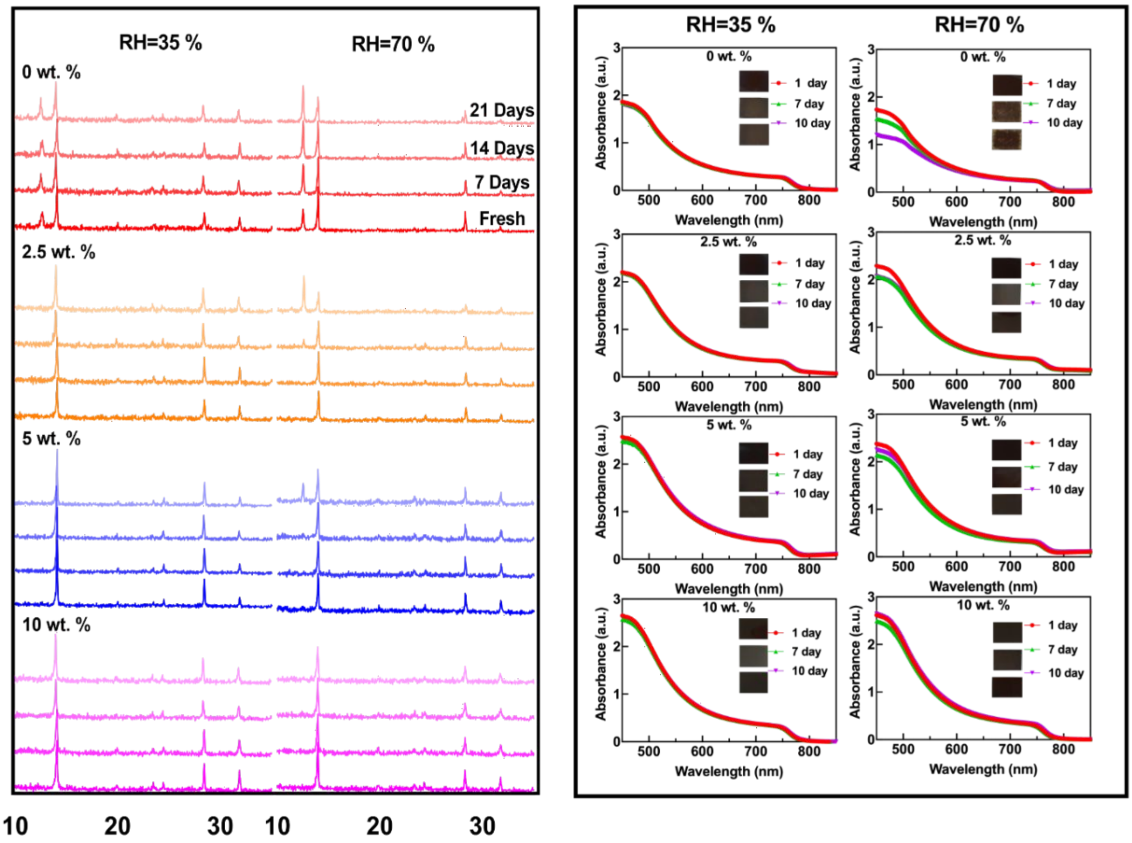

Figure S4. XRD patterns (left) and the UV-VIS-NIR absorbance spectra changes (right) of MAPI films with various concentrations of pHEMA (0 – 10 wt. %), aged at 35 % RH (ambient) and 70 % RH.

Film appearance during degradation

As shown in Fig. S5, the change in appearance of the film can be observed by tracking the degradation of the film in different humidities. In about 35 % RH at RT, the pure MAPI film began to turn pale around the fifth or sixth day, marking the initial decomposition of the perovskite; while those incorporated with pHEMA maintained their initial colour throughout the observation window, due to the passivating role of pHEMA. In a more severe environment of about 70 % RH at RT, the reference sample on the fifth day showed a yellow PbI_2 phase, indicating that the MAPI film was severely degraded. The appearance of the pHEMA-incorporated MAPI film remained close to the fresh sample, with no obvious change visible to the naked eye, suggesting excellent stability.

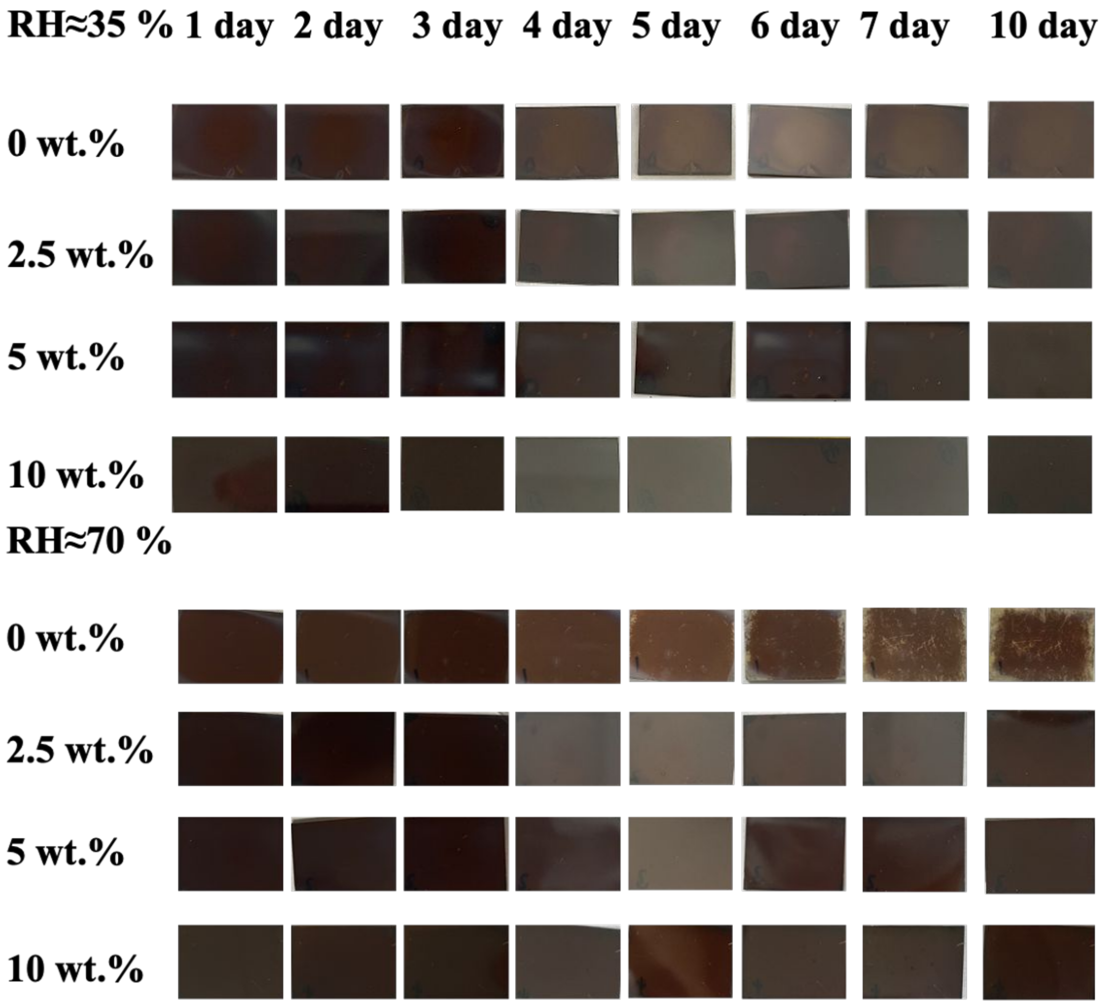

Figure S5. The change in appearance of perovskite films as a function of time after exposure to ambient air at roughly 35 % and 70 % RH.

Thermal decomposition under UHV conditions

0 wt. %			
Temperature	I/Pb^{2+}	N/Pb^{2+}	Pb^0/Pb^{2+}
RT	3.0 ± 0.1	0.7 ± 0.1	-
100 °C	2.9 ± 0.1	0.6 ± 0.1	-
150 °C	2.5 ± 0.1	0.2 ± 0.1	0.02 ± 0.01
180 °C	2.1 ± 0.1	-	0.26 ± 0.01

Table S4. Elemental ratios in a 0 wt. % MAPI film as a function of temperature. Values were calculated from core level XPS spectra displayed in Fig. 1.

5 wt. %			
Temperature	I/Pb^{2+}	N/Pb^{2+}	Pb^0/Pb^{2+}
RT	2.3 ± 0.1	0.7 ± 0.1	-
100 °C	2.4 ± 0.1	0.6 ± 0.1	-
150 °C	2.2 ± 0.1	0.3 ± 0.1	-
180 °C	2.1 ± 0.1	-	0.05 ± 0.01

Table S5. Elemental ratios in a 5 wt. % MAPI-pHEMA film as a function of temperature. Values were calculated from core level XPS spectra displayed in Fig. 1.

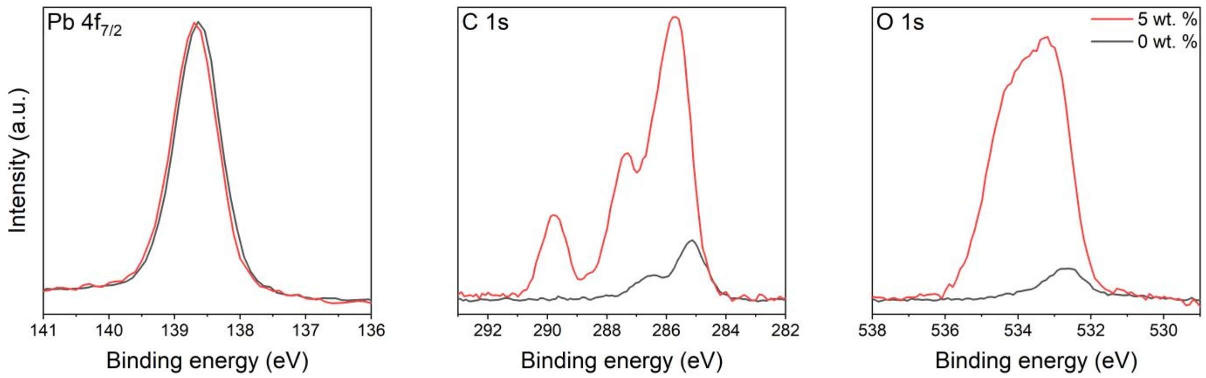

Figure S6. Overlaid Pb $4f_{7/2}$, C 1s and O 1s (left to right) high-resolution core level XPS spectra from 0 wt. % (black) and 5 wt. % (red) MAPI-pHEMA films, measured at room temperature (RT).

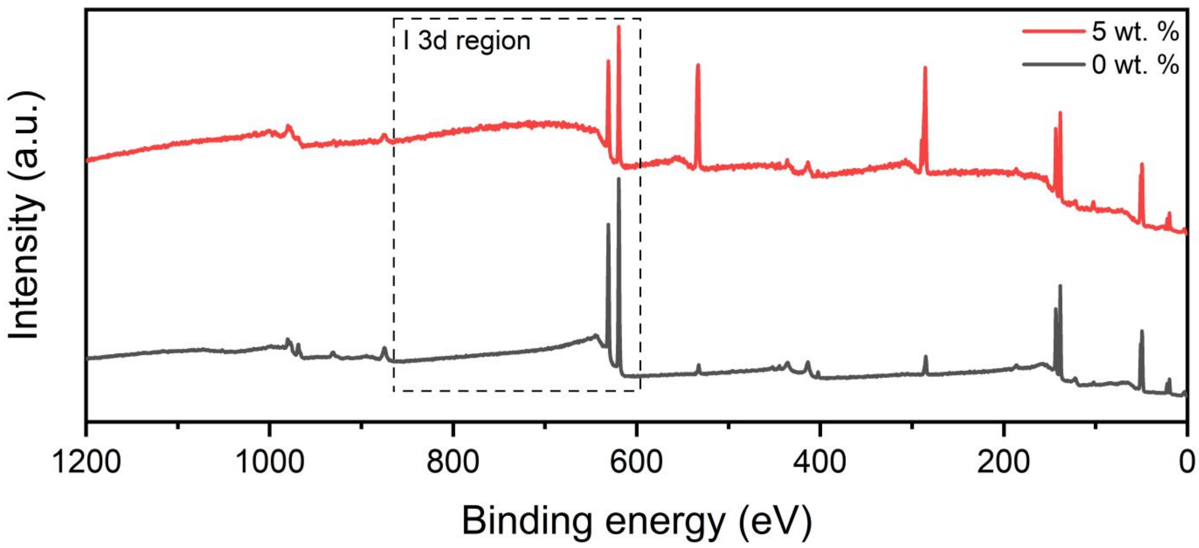

Figure S7. XPS survey spectra of pristine 0 wt. % and 5 wt. % MAPI-pHEMA films. The region following the I 3d doublet peak used for background modelling is marked.

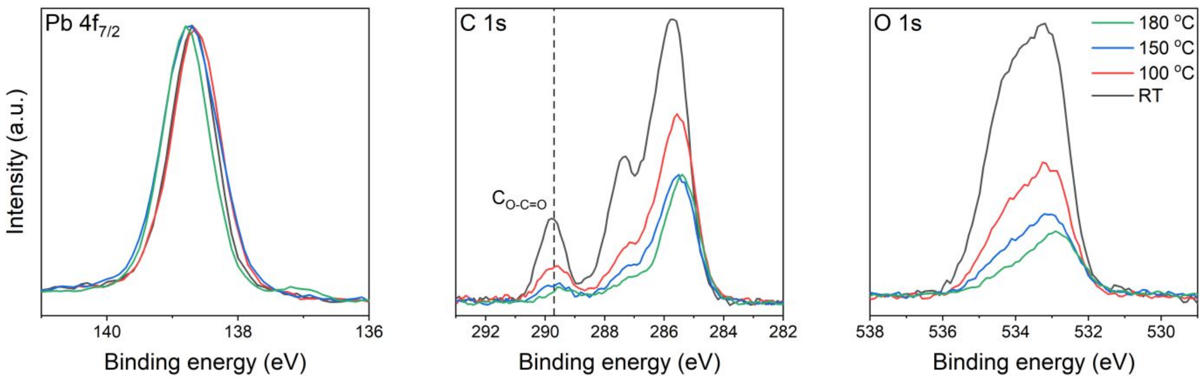

Figure S8. Overlaid Pb $4f_{7/2}$, C 1s and O 1s (left to right) high-resolution core level XPS spectra of a 5 wt. % MAPI-pHEMA film at various stages of heating (RT – 180 °C). The peak at 289.7 eV BE (labelled $C_{O\text{-}C=O}$) is assigned to the O-C=O bonding environment in pHEMA. The intensity of this peak was used to track the change in concentration of pHEMA at the surface of the sample. It is important to note that this peak intensity does not correspond to the total concentration of pHEMA, only the concentration of O-C=O bonds.

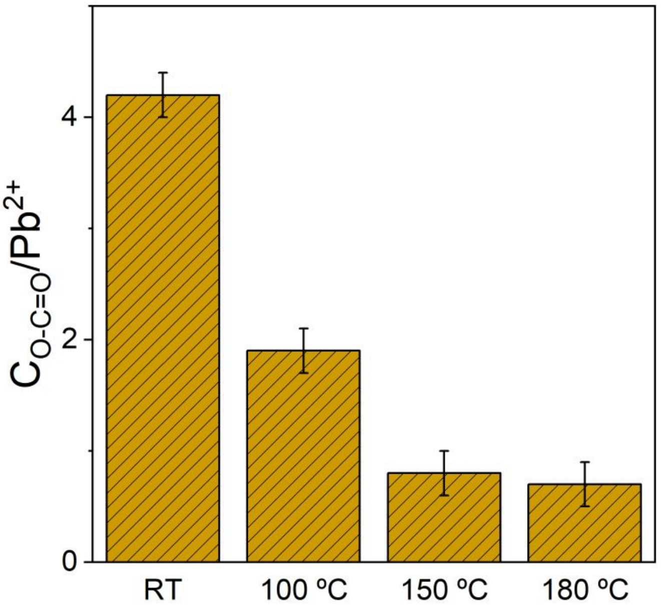

Figure S9. Surface ratio of $C_{O\text{-}C=O}/Pb^{2+}$ as a function of sample temperature, determined from the intensity of peaks fitted to the Pb^{2+} and $C_{O\text{-}C=O}$ features in the high-resolution core level XPS spectra of a 5 wt. % MAPI-pHEMA film.

Estimation of overlayer-corrected nominal stoichiometry

The presence of a pHEMA-rich layer at the surface of the 5 wt. % films, observed in both XPS and HAXPES measurements, requires us to reconsider the stoichiometry we expect to observe in XPS and HAXPES of the pHEMA-incorporated films. For a fixed photon energy (as here), the KE of photoelectrons from the core levels used to calculate the elemental ratios is not constant, and hence the sampling depth is different for each element measured. For a homogenous sample, this can usually be easily corrected for during analysis. However, in the presence of an overlayer, photoelectrons from higher BE core levels, and therefore lower KE, experience greater attenuation, leading to an underestimation of elemental concentration in comparison with signals from lower BE core levels.

To account for the varying loss of signal due to the pHEMA overlayer we can approximate the sample to a 2-layer system, and use the flux attenuation expression shown in Equation S1:

$$I = I_0 \int_d^{\infty} exp\left(-\frac{x}{\lambda}\right) dx = I_0 \lambda \exp\left(-\frac{d}{\lambda}\right) \quad \textbf{(S1)}$$

Here, I and I_0 represent the observed and emitted photoelectron intensity from a given core level, d is the effective pHEMA overlayer thickness (calculated as *ca.* 3 nm at RT using inelastic background modelling of both XPS and HAXPES) and λ is the IMFP of photoelectrons from the given core level (approximated to the IMFP through pHEMA, *i.e.* we assume a uniform overlayer containing pHEMA only, overlying a bulk consisting of stoichiometric MAPI only). Equation 2 shows the intensity ratio of two core levels, from elements x and y.

$$\frac{I_x}{I_y} = \frac{N_x \sigma_x}{N_y \sigma_y} T_{rel} = \frac{N_{x,0} \sigma_x}{N_{y,0} \sigma_y} T_{rel} \frac{\lambda_x \exp\left(-\frac{d}{\lambda_x}\right)}{\lambda_y \exp\left(-\frac{d}{\lambda_y}\right)} \quad \textbf{(S2)}$$

Here, $N_{x,0}/N_{y,0}$ is the nominal stoichiometric ratio for elements x and y, and N_x/N_y is the corrected ratio, taking into account the attenuation of signal from both core levels by the overlayer. σ represents the photoemission cross section for each core level and T_{rel} is a factor correcting for the change in analyser transmission function between the KE ranges of the peaks compared. In Equation S2, the σ and T_{rel} factors cancel to give an equation for corrected stoichiometric ratio, shown in Equation S3.

$$\frac{N_x}{N_x} = \frac{N_{x,0}}{N_{y,0}} \frac{\lambda_x \exp\left(-\frac{d}{\lambda_x}\right)}{\lambda_y \exp\left(-\frac{d}{\lambda_y}\right)} \quad \textbf{(S3)}$$

Corrected stoichiometric ratios (*i.e.* those we expect to observe experimentally) for MAPI with a 3 nm pHEMA overlayer for both XPS (hν = 1486.6 eV) and HAXPES (hν = 9250 eV) are displayed in Table S6.

Core level	hν (eV)	KE difference (eV)	Nominal	Corrected
I 3d/Pb 4f	1486.6	- 481	3	1.5
N 1s/Pb 4f	1486.6	- 264	1	0.7
I $2p_{3/2}$/Pb $3d_{5/2}$	9250	- 2073	3	2.0
I $3p_{3/2}$/Pb $4p_{3/2}$	9250	- 228	3	2.9
N 1s/Pb 4d	9250	+ 11	1	1.0

Table S6. Corrected elemental ratios for MAPI due to a 3 nm pHEMA overlayer, calculated using Equation S3. In all cases, IMFP through pHEMA was used as an approximate value. Negative KE difference represents numerator KE < denominator KE.

***Ex situ* moisture-induced decomposition**

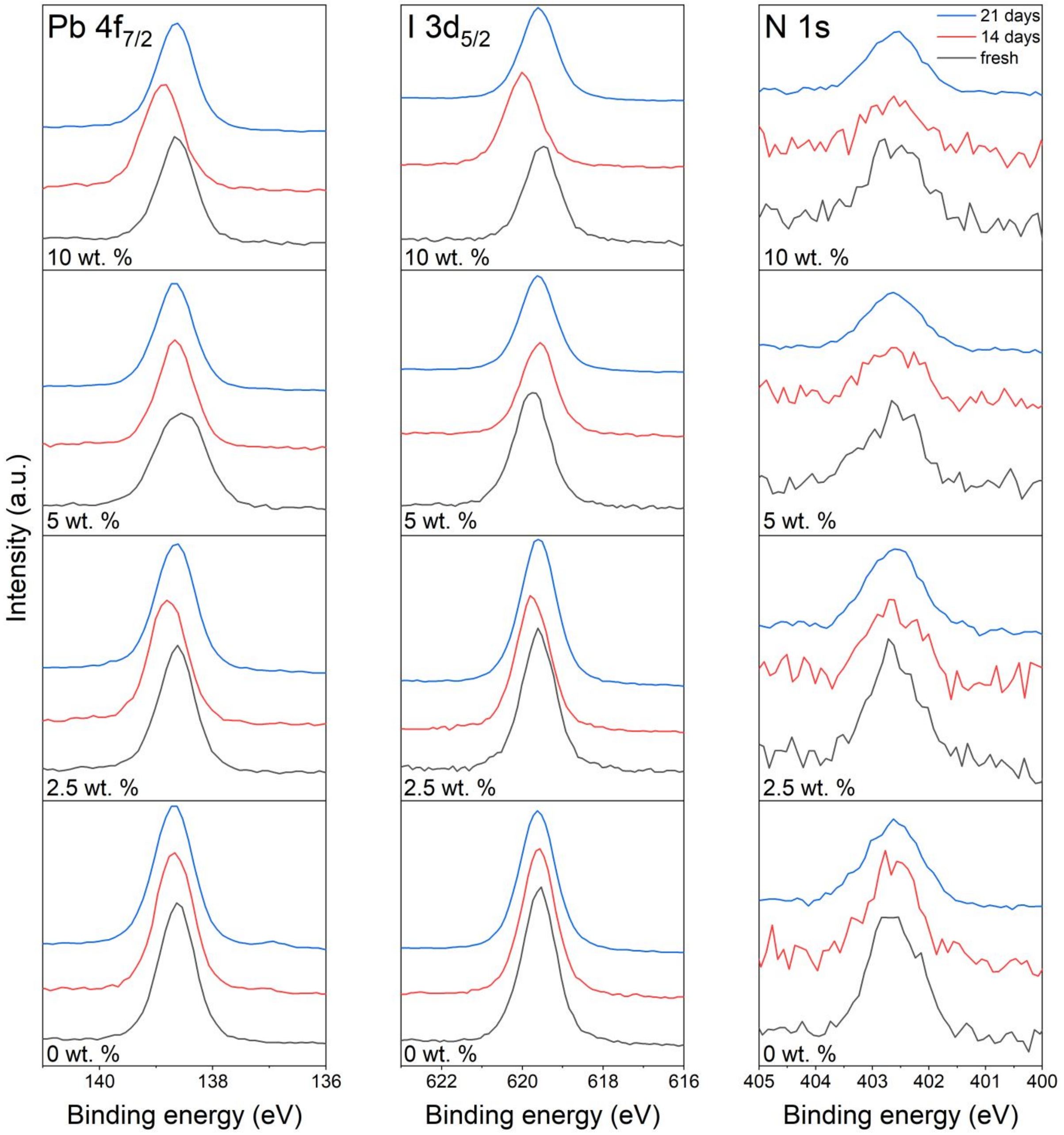

Figure S10. Pb $4f_{7/2}$, I $3d_{5/2}$ and N 1s (left to right) high-resolution core level XPS spectra of MAPI films containing various concentrations of pHEMA (0 – 10 wt. %). Samples were measured following fabrication (fresh), and after 14 and 21 days of storage in 35 % RH.

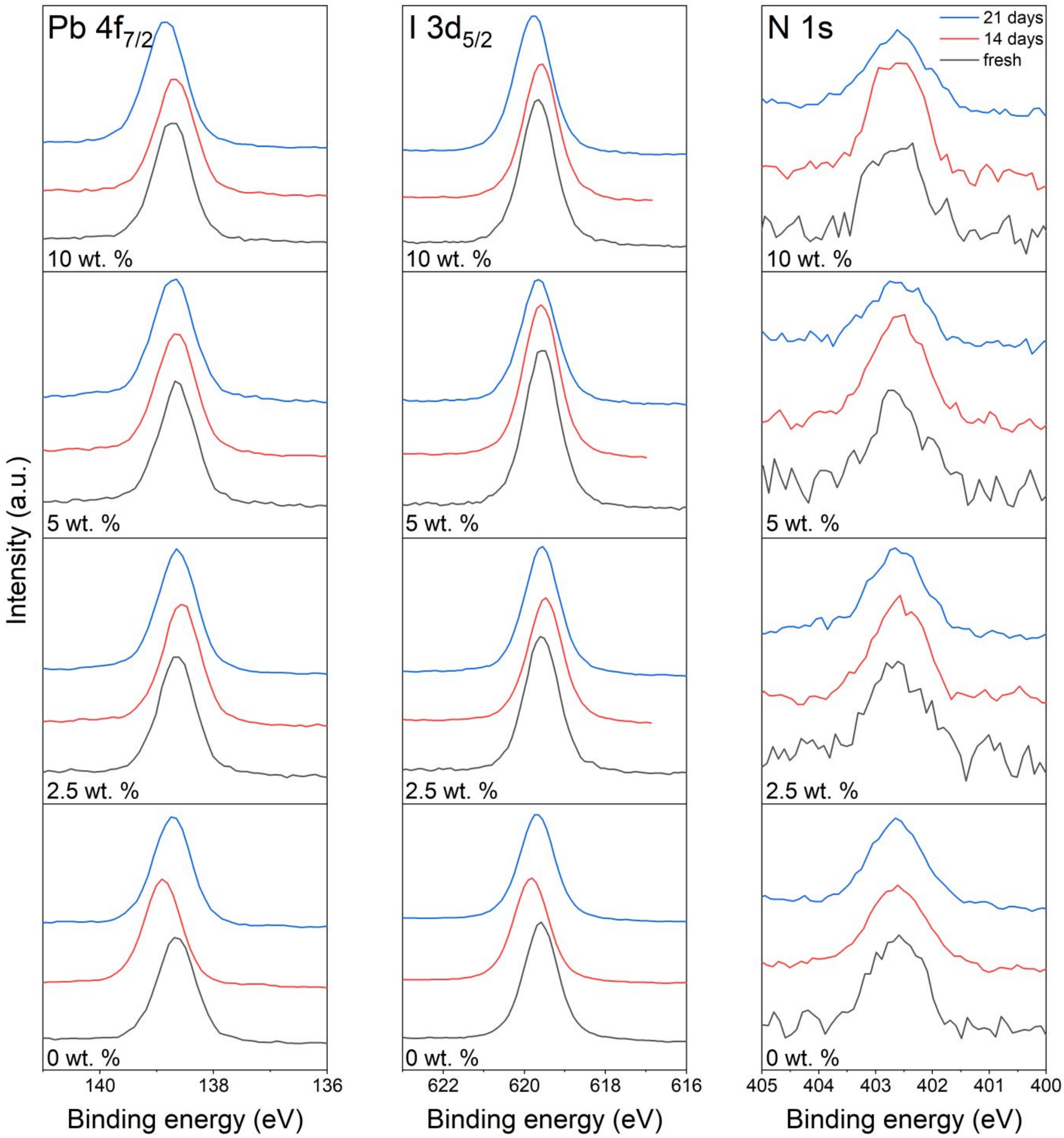

Figure S11. Pb $4f_{7/2}$, I $3d_{5/2}$ and N 1s (left to right) high-resolution core level XPS spectra of MAPI films containing various concentrations of pHEMA (0 – 10 wt. %). Samples were measured following fabrication (fresh), and after 14 and 21 days of storage in 70 % RH.

To provide further insight into the effect of pHEMA concentration on the stability of MAPI, XPS was performed on MAPI films containing various concentrations of pHEMA (0 – 10 wt. %). Data were acquired following sample fabrication (fresh), and after 14 and 21 days of storage *ex situ* in containers with controlled humidity. Figs. S10 and S11 display Pb $4f_{7/2}$, I $3d_{5/2}$ and N 1s high-resolution core level XPS spectra acquired from films with various concentrations of pHEMA, stored in 35 % and 70 % RH respectively. No significant core level shifts are observed in samples stored at 35 % RH after 21 days; however, the 0 wt. % (pure MAPI) and 10 wt. % films each experience a +0.1 eV BE shift in Pb $4f_{7/2}$ and I $3d_{5/2}$ peaks position following 21 days at 70 %. However, these shifts were found to differ between

different spots on the sample surface, so may only be evidence of an area of localized degradation, rather than increased degradation across the whole sample.

Elemental concentrations were determined from the intensity of peaks fitted to the core level spectra and are displayed in Figure S12, as a function of pHEMA concentration and moisture exposure. Little change in the I/Pb^{2+} ratio and only a slight reduction in the N/Pb^{2+} ratio is observed for all samples at both humidities. However, the concentration of Pb^0 appears to increase more rapidly in the MAPI sample compared to all concentrations of pHEMA, as observed in our *in situ* degradation studies.

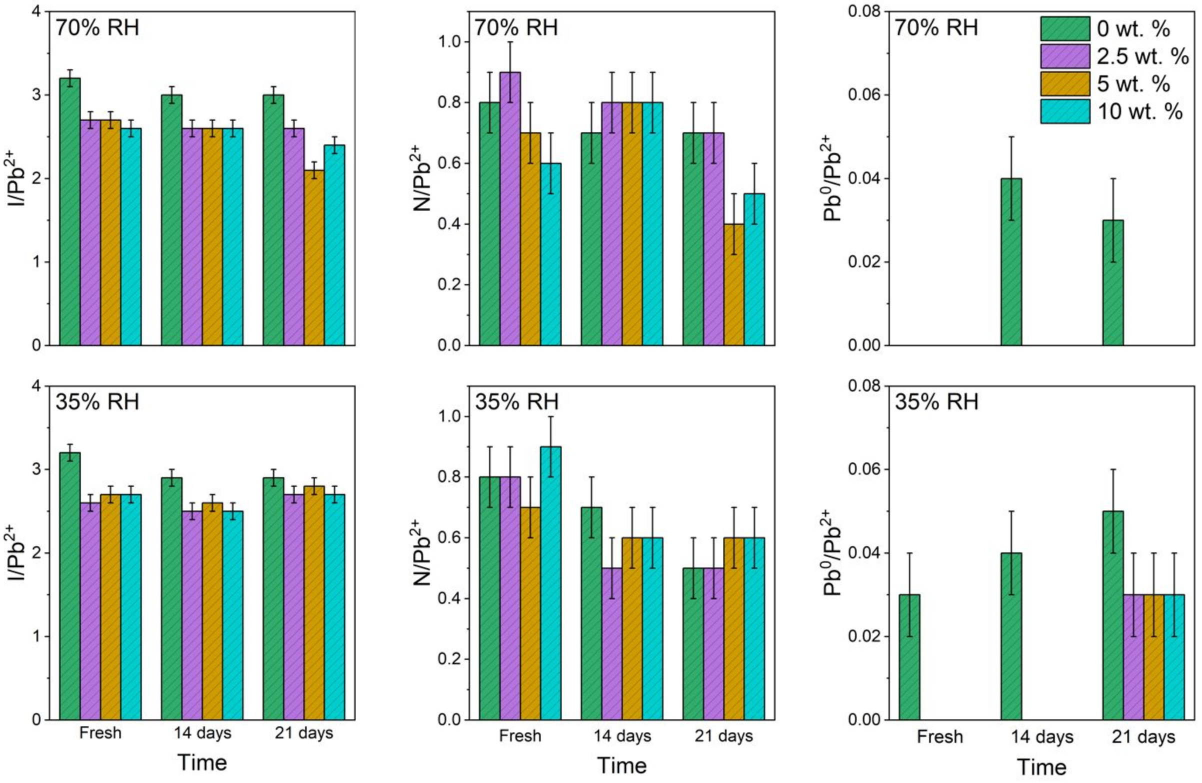

Figure S12. Surface stoichiometry of MAPI films containing various concentrations of pHEMA (0 - 10 wt. %) as a function of sample temperature, determined from fitted XPS peak intensities. I, N and Pb^0 concentrations with respect to Pb^{2+} (left to right) are displayed. The nominal stoichiometry of MAPI is 1 : 3 : 1 (Pb^{2+} : I : N).

Angle-resolved HAXPES depth profiling

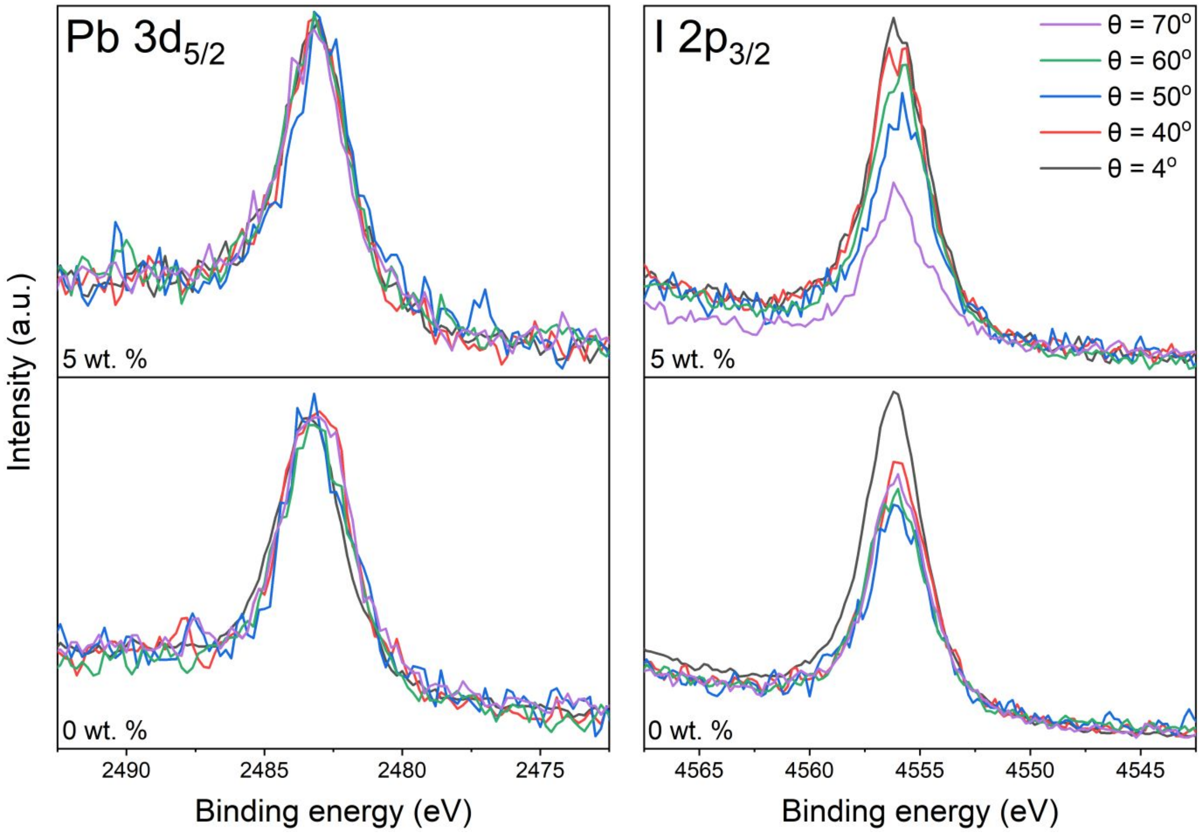

Figure S13. Pb $3d_{5/2}$ and I $2p_{3/2}$ (left and right) high-resolution core level HAXPES spectra of fresh 0 wt. % (bottom) and 5 wt. % (top) MAPI-pHEMA films, at various electron emission angles relative to the surface normal (θ = 4° – 70°). Increasing emission angle corresponds to decreasing sampling depth, as outlined by eqn (3) in the main text.

Emission angle (°)	Sampling depth (nm)	0 wt. %	5 wt. %
4	24	3.7 ± 0.3	2.7 ± 0.3
40	18	2.7 ± 0.3	2.4 ± 0.3
50	15	2.6 ± 0.3	2.0 ± 0.3
60	12	2.5 ± 0.3	2.2 ± 0.3
70	8	2.7 ± 0.3	1.5 ± 0.3

Table S7. I/Pb^{2+} ratios of 0 wt. % and 5 wt. % films as a function of electron emission angle. Values were calculated from core level HAXPES spectra displayed in Fig. S9. Sampling depth was approximated using the IMFP of I $2p_{3/2}$ photoelectrons passing through MAPI (8 nm), and calculated with eqn (3) in the main text.

Emission angle (°)	Sampling depth (nm)	0 wt. %	5 wt. %
4	42	1.5 ± 0.3	1.5 ± 0.3
40	32	1.5 ± 0.4	1.6 ± 0.5
50	27	1.8 ± 0.6	2.3 ± 0.7
60	21	1.5 ± 0.6	2.5 ± 0.7
70	14	0.5 ± 0.3	2.7 ± 0.7

Table S8. N/Pb^{2+} ratios of 0 wt. % and 5 wt. % films as a function of electron emission angle. Values were calculated from core level HAXPES spectra. Sampling depth was approximated using the IMFP of N 1s photoelectrons passing through MAPI (14 nm), and calculated with eqn (3) in the main text.

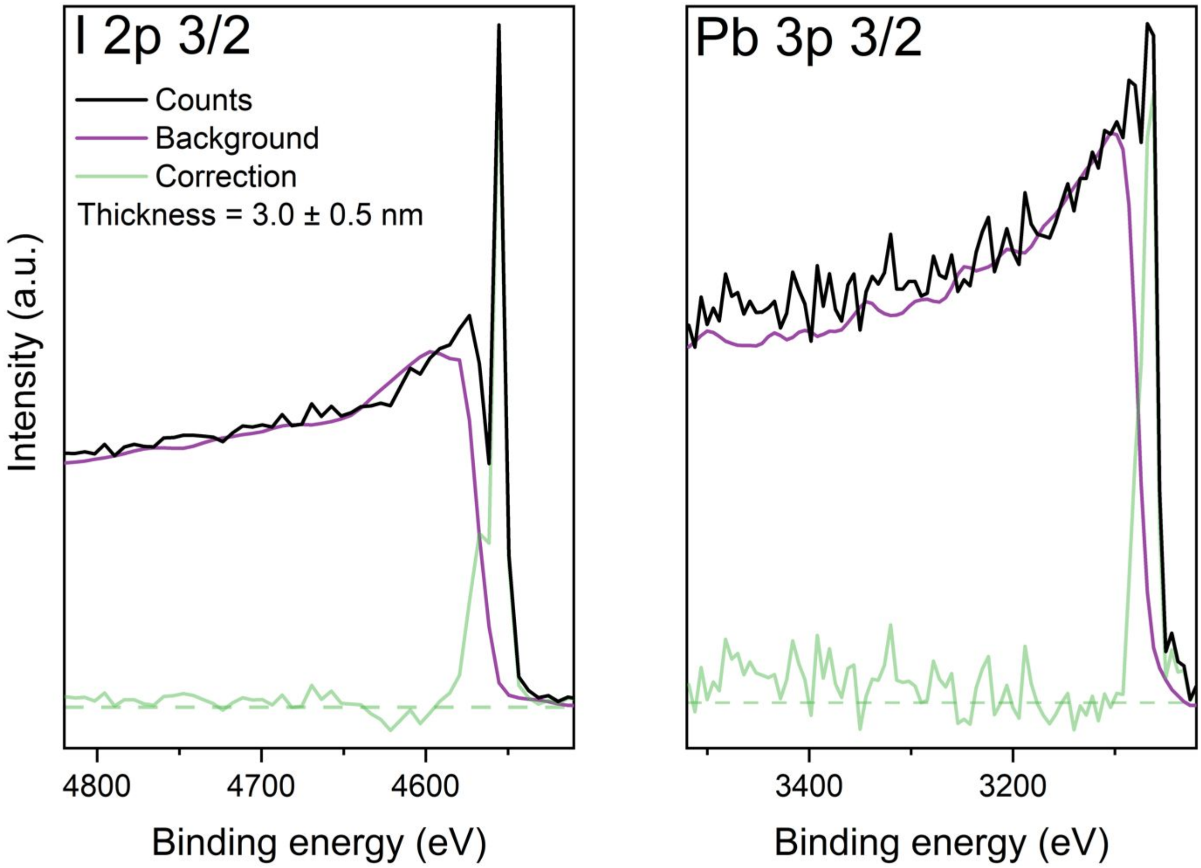

Figure S14. Inelastic background fits to the I $2p_{3/2}$ and Pb $3p_{3/2}$ regions of the θ = 4° HAXPES survey scan from a 5 wt. % pHEMA-MAPI sample, generated using the QUASES-Tougaard software.[5, 6] The purple line corresponds to a simulated inelastic background from MAPI topped with a 3 nm overlayer of pHEMA, and the black line shows the true background.

Self-healing hydrogel network

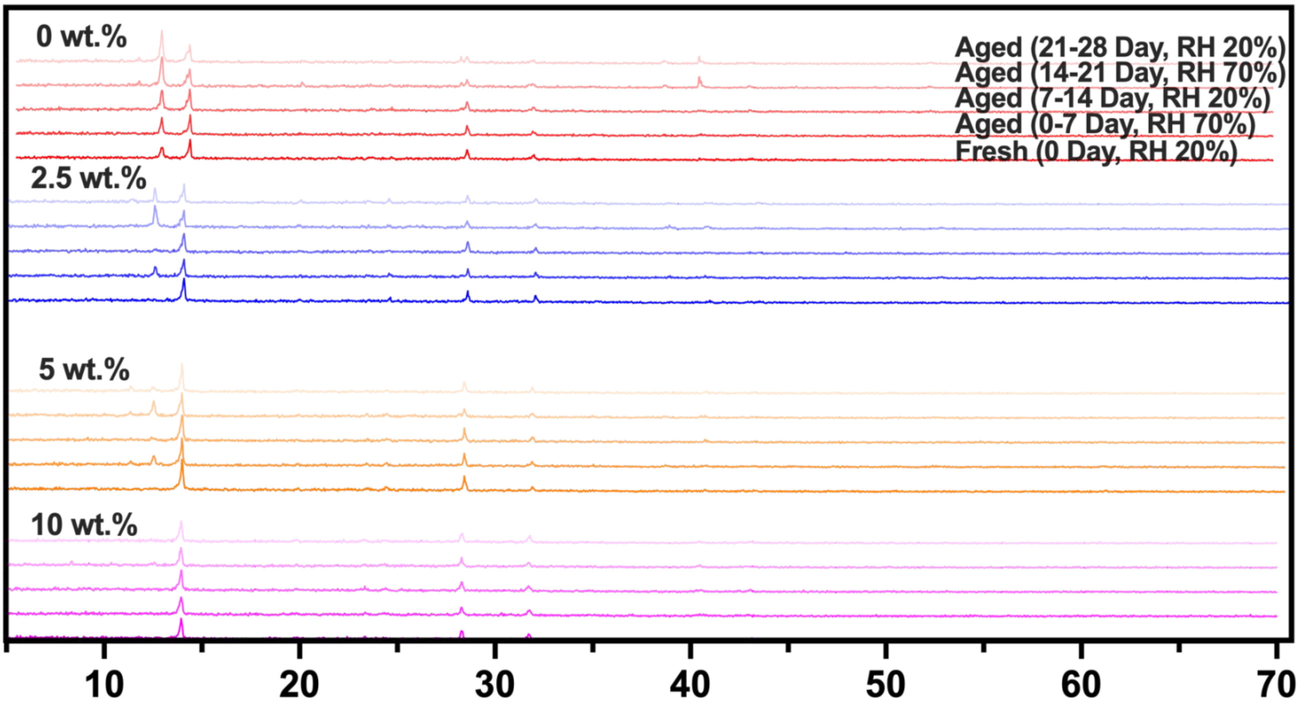

Figure S15. XRD patterns of the various aging perovskite films kept in varying humidity (fresh, 0 - 7 days at 70 % RH, 7 - 14 days at 20 % RH, 14 - 21 days at 70 % RH and 21 - 28 days at 20 % RH)

References

1. W. Huang, F. Huang, E. Gann, Y. B. Cheng and C. R. McNeill, *Advanced Functional Materials*, 2015, **25**, 5529-5536.
2. S. Urquhart and H. Ade, *The Journal of Physical Chemistry B*, 2002, **106**, 8531-8538.
3. K. Hermann, L. G. M. Pettersson, M. E. Casida, C. Daul, A. Goursot, A. Koester, E. Proynov, A. St-Amant, D. R. Salahub and V. Carravetta, *StoBe-DeMon 3.3*, 2014.
4. C. M. Sterling, C. Kamal, G. J. Man, P. K. Nayak, K. A. Simonov, S. Svanstrom, A. García-Fernández, T. Huthwelker, U. B. Cappel and S. M. Butorin, *The Journal of Physical Chemistry C*, 2021, **125**, 8360-8368.
5. S. Tougaard, *Journal of Vacuum Science & Technology A: Vacuum, Surfaces, and Films*, 2021, **39**, 011201.
6. S. Tougaard, *QUASES: Software for Quantitative XPS/AES of Surface Nanostructures*, 1994–2018, **Ver. 7.00**.
